# Native bees of high Andes of Central Chile (Hymenoptera: Apoidea): biodiversity, phenology and the description of a new species of *Xeromelissa* Cockerell (Hymenoptera: Colletidae: Xeromelissinae)

**DOI:** 10.7717/peerj.8675

**Published:** 2020-02-28

**Authors:** Patricia Henríquez-Piskulich, Cristian A. Villagra, Alejandro Vera

**Affiliations:** 1 Instituto de Entomología, Universidad Metropolitana de Ciencias de la Educación, Santiago, Región Metropolitana, Chile; 2 Departamento de Biología, Universidad Metropolitana de Ciencias de la Educación, Santiago, Región Metropolitana, Chile

**Keywords:** Apoidea, Hotspot, Neotropics, Taxonomy, Underrepresented zones, Xeromelissinae

## Abstract

High-altitude ecosystems are found in mountain chains and plateaus worldwide. These areas tend to be underrepresented in insect biodiversity assessments because of the challenges related to systematic survey at these elevations, such as extreme climatic and geographic conditions. Nonetheless, high-altitude ecosystems are of paramount importance because they have been seen to be species pumps for other geographic areas, such as adjacent locations, functioning as buffers for population declines. Moreover, these ecosystems and their biodiversity have been proposed to be fast-responding indicators of the impacts caused by global climate change. Bees have been highlighted among the insect groups that have been affected by these problems. This work used bees as a proxy to demonstrate and reinforce the importance of systematic surveys of high-altitude ecosystems. Here, field collections were undertaken and an updated review was conducted for the native bee biodiversity of the high-altitude ecosystem found at the Andes system of central Chile, including the phenological trends of these insects during the flowering season. Of the 58 species that have been described for this location, we were able to confirm the occurrence of 46 of these species as a result of our sampling. In addition, thanks to these recent collections, a new species of *Xeromelissa* Cockerell is described in the present work. These findings highlight the need for further high-altitude insect surveys of this biome, which include both temporal and spatial complexity in their design, to allow for accurate assessment of bee species diversity and compositional changes in these mountain regions.

## Introduction

From a global point of view, bees are among the most well-known insect groups to date; over 20,000 species and their distributions have been described (Ascher & Pickering, 2019). However, understudied areas of the planet still exist and there are considerable knowledge gaps on bee species diversity and biology (Freitas et al., 2009). This is a matter especially important, due to the current global pollinator declines and conservation needs (Potts et al., 2010). For instance, in the case of Chile, a country with a biodiversity hotspot in its central region (Myers et al., 2000), most recent information comes from original species descriptions and has been contributed by a rather scarce number of researchers (e.g., Ferrari, 2017; González-Vaquero & Galvani, 2016; Monckton, 2016; Packer & Dumesh, 2014; Roig-Alsina, 1991; Roig-Alsina, 1999; Rojas, 2001; Rojas & Toro, 2000; Ruz & Toro, 1983; Toro & Moldenke, 1979; Toro & Rodríguez, 1998; Toro & Ruz, 1969; Vivallo, 2009; Vivallo, 2013; Vivallo, 2014; Vivallo, Zanella & Toro, 2003, among others). Also, within Apoidea, some taxonomic groups receive more attention than others. As a result, there are still wide gaps in regards to the number of species, their temporal and spatial patterns, ecological associations, and the links between males and females.

High-altitude ecosystems could be among the least studied areas concerning bee biodiversity, due to the difficulty of accessing and sampling these locations, as well as a rather restricted time frame for conducting samplings due to snowfall. Mountain and plateau systems cover approximately 22% of Earth’s land surface distributed in all climatic zones (Manuelli, Hofer & Springgay, 2019), often harboring a high biodiversity (Hoorn et al., 2013) and comprising different ecoregions, such as montane grasslands, scrublands, and tundra (Öztürk et al., 2015; WWF, 2019). Furthermore, each of these have communities with an assemblage of species with crucial contributions to global diversity (Paknia & Pfeiffer, 2011; González-Reyes, Corronca & Rodriguez-Artigas, 2017). Ecological regions adjacent to high-altitude ecosystems may also share high proportions of species in common, and could also act as filters for the distribution and/or exchange of other species (González-Reyes, Corronca & Rodriguez-Artigas, 2017). This means they could limit the distribution of certain species, and at the same time, support a higher diversity of shared generalist species. They could also shape mountain communities and function as “species pumps”; supplying other ecosystems with resources and organism diversity by dispersal events (Sedano & Burns, 2010).

To the best of our knowledge, Chile has a 70% endemism rate in native bees (Montalva & Ruz, 2010). Furthermore, the central regions of this country are part of a global biodiversity hotspot (Myers et al., 2000). Considering these facts, it is plausible to hypothesize that this portion of the High Andes could be a reservoir for an important and diverse group of native bee species with potentially key ecological roles (Wilson et al., 2010; Tucker & Rehan, 2017). Regarding bee biodiversity, this zone has been scarcely studied, and the available literature is highly biased towards the study of the role that these insects play as pollinators (Arroyo, Primack & Armesto, 1982; Camousseight & Barrera, 1998; Murúa, Cisterna & Rosende, 2014). Only recently the focus has been centered on other aspects, such as the potential conservation threats for these species (Henríquez-Piskulich et al., 2018).

It is possible that Chilean mountain ranges may have a considerable number of undescribed bee species if we take into account the high bee diversity found in other high-altitude ecological regions of the Andes (González & Engel, 2004). Additionally it has been discovered that anthropogenic climate change is related to phenological shifts in bee species, with the consequence of a mismatch between the ancestral activity period of bee species and flowering period (Memmott et al., 2007; Hegland et al., 2009). For these reasons, it is apparent that there is an urgent need for further research, considering present threats such as global warming and other human-derived disturbances and the need for planet-scale insect conservation (Gottfried et al., 1999; Öztürk et al., 2015; Melo et al., 2017; Sánchez-Bayoa & Wyckhuys, 2019). Given that high-altitude ecosystems are experiencing the earliest and greatest impacts of climate change (Ruiz et al., 2008; Palomo, 2017), the knowledge regarding adult phenology of mountain bee species is also critical.

In this article, we provide an updated list of native bee species and the associated adult phenology at a genus level, obtained from recent field collections in the high Andean ecosystem of Farellones in central Chile, and compare these collections with previously published lists. Furthermore, we describe a new species of *Xeromelissa* Cockerell (Xeromelissinae) for this location. The latest major revision of the Chilean Xeromelissinae was done by Toro & Moldenke (1979), and the subfamily was later subjected to phylogenetic analysis, and classificatory review by Packer (2008). Although distributed primarily in Chile, *Xeromelissa* is also found in Peru and Argentina, with 21 described species (Moure & Urban, 2012) and potentially with a total of approximately 40 species (Packer, 2019). We discuss these findings, emphasizing the need for further entomological research in high-altitude areas where there are still taxa in need of being discovered and described.

## Materials & Methods

For the list of bees in this current work, 1,091 specimens were re-examined, which had been previously collected in the subandean vegetational belt of Farellones (33°18′00.5″S 70°15′13.3″W) in the Andes mountains of central Chile for the purposes of a previous study in the same area (Henríquez-Piskulich et al., 2018). Bees were sampled from eight sites of 80 × 80 m, following the methods of Fortel et al. (2014), using pan traps and insect nets. Pan traps were left to work at each site from 9:00 to 17:00 (Westphal et al., 2008), while active net sampling was done one hour during the morning (9:00–12:00) and one hour during the afternoon (15:00–17:00). Specimens were collected once a month for two seasons: the first in December 2016, January and February 2017 (season 1: 2016/2017), and the second in November and December 2017, January and February 2018 (season 2: 2017/2018). *Xeromelissa farellones* Toro & Moldenke was also added to the list, which was collected during field work of January 2019. Because *Bombus dahlbomii* Guérin-Méneville is an endangered and conspicuous species (Morales et al., 2016), they were only collected to take into account the relative abundance and after the one hour sampling period they were all released. All specimens collected were identified using existing keys (Toro & Ruz, 1969; Sielfeld, 1973; Toro & Moldenke, 1979; Ruz & Toro, 1983; Chiappa, Rojas & Toro, 1990; Roig-Alsina, 1991; Roig-Alsina, 1999; Toro & Rodríguez, 1998; Rojas & Toro, 2000; Rojas, 2001; Vivallo, Zanella & Toro, 2003; Durante, Abrahamovich & Lucia, 2006; Vivallo, 2009; Vivallo, 2013; Vivallo, 2014; González-Vaquero & Galvani, 2016; Monckton, 2016; Ferrari, 2017). Later, this list was compared to previously published works done in the same location regarding native bee species between 1979 and 1981 (Arroyo, Primack & Armesto, 1982; Camousseight & Barrera, 1998). Genera and species names listed in the aforementioned papers were revised according to currently accepted nomenclature. The specimens that were identified only to genus were not included in the analysis to facilitate comparison among surveys. Moreover, the specimens studied by Camousseight & Barrera (1998), available in the entomological collection of the Museo Nacional de Historia Natural, Santiago de Chile (MNHNC), were re-examined. Table S1 shows the full list of species present for each survey. The coordinates of the site locations, the abundance of each species and the exact dates the specimens were collected for the survey done by Henríquez-Piskulich et al. (2018) can be found in the supporting dataset.

In order to know about the abundance of adults from each family (i.e., Andrenidae, Colletidae, Halictidae, Megachilidae, and Apidae) found in the recent collections of this high-altitude area, a general Chi Square test followed by a Multiple Proportion Comparison Test was applied (Zar, 1999). To evaluate the occurrence of adult phenology of the different genera, circular statistical analysis was used for field season 2017-2018 collections. The first season wasn’t included in the analysis to avoid biases because it wasn’t possible to sample in November due to bad weather conditions. For each genus the following parameters were calculated: mean direction (theta), concentration of collection (R bar), circular spread (delta) and the concentration of collections using Von Mises distribution kappa parameter (Fisher, 1993; Dos Santos et al., 2017; Frías-Lasserre & Villagra, 2019). Rayleigh (z) test was used to test uniform distribution goodness-of-fit assessing the prevalence of different bee families along the 12-month year clock; which evaluated the significance of mean per month of collection for each group. An even distribution of bee family collections around the year clock was considered as a null hypothesis. Alternatively, it was hypothesized that different families were found concentrated showing a temporal pattern (Morellato, Alberti & Hudson, 2010). Statistics were run with Minitab, LLC^®^ and NCSS^®^ software.

To evaluate the difference of the recent collections with the other two previously published works (Arroyo, Primack & Armesto, 1982; Camousseight & Barrera, 1998), the similarity of the bee collections obtained between different studies were compared using the Jaccard similarity index (JSI) (Jaccard, 1900), expressed as:

\begin{eqnarray*}\mathrm{JSI}=[\mathit{a}/(\mathit{a}+\mathit{b}+\mathit{c})]\ast 100 \end{eqnarray*}

Where ***a*** is the number of species in common to two given groups, ***b*** is the number of species solely found in one of the groups and ***c*** is the number of those that can only be found in the other. This index, expressed as a percentage, uses the presence–absence relationship between the numbers of species in common in two groups of samples and studies its relation with the total number of species identified, which reflects the percentage of similarity between the compared groups (Taylor, Kent & Coker, 1993).

For the description of the new *Xeromelissa* species, body measurements were taken using the software Leica Application Suite version 3.4.0 with a Leica MC170 HD camera mounted on a Leica S8 APO stereomicroscope. Male terminalia were cleared in 5% NaOH for five hours and then stored in glycerin. Terminology for morphology follows Packer & Genaro (2007) and for surface sculpture, Harris (1979). Measurements of body length, head width, thorax width, and forewing length were recorded in millimeters (mm). Images from scanning electron microscopy (SEM) were taken with a Hitachi TM3000 Benchtop. All specimen images were taken with Cognisys StackShot 3X system, a Canon EOS 60D camera, a Canon MP-E 65 mm lens and a Canon EF 2X III extender. Image slices were assembled using the stacking software Zerene Stacker version 1.04. Images were later edited, cropped, and scale bars added using Adobe Photoshop Lightroom Classic CC version 7.1 and Adobe Photoshop CC version 2017. We deposited all type specimens of the new species in the MNHNC, as well as a representative sample of the species listed in this study. We used *X. farrellones* for comparison purposes because it shares its distribution with the new species described in this work. Furthermore, we studied the original descriptions and the subsequent works of every species. The characters of diagnosis of the described species have been based primarily on the male terminalia, which presents characteristics that differentiate it from previously described species, where reproduction incompatibility linked to the “lock-and-key” hypothesis is inferred. The new species described in this work differs in these characteristics, which have similarly been used to define species in works by previous authors. Other characters of the integument and of coloration in both sexes, independently from the terminalia, back up this diagnosis. We also consulted reference material from the Museo Nacional de Historia Natural Santiago de Chile and the entomological collection of bees of the Pontificia Universidad Católica de Valparaíso, where there is material identified by the original authors of several species.

The electronic version of this article in Portable Document Format (PDF) will represent a published work according to the International Commission on Zoological Nomenclature (ICZN), and hence the new names contained in the electronic version are effectively published under that Code from the electronic edition alone. This published work and the nomenclatural acts it contains have been registered in ZooBank, the online registration system for the ICZN. The ZooBank LSIDs (Life Science Identifiers) can be resolved and the associated information viewed through any standard web browser by appending the LSID to the prefix http://zoobank.org/. The LSID for this publication is: urn:lsid:zoobank.org:pub:CAF131C8-3C76-49F0-87F9-76402590D913. The online version of this work is archived and available from the following digital repositories: PeerJ, PubMed Central and CLOCKSS.

## Results

### Native bee survey comparison

The sum of the three published works for Farellones (Arroyo, Primack & Armesto, 1982; Camousseight & Barrera, 1998; and the present study) reaches a total of 58 species for this high-altitude ecosystem (Table 1). Based on the current survey, it was possible to identify 46 species. When comparing this with the two previous studies, 17 species were shared with Arroyo, Primack & Armesto (1982) with a JSI of 34%, and eight with Camousseight & Barrera (1998), reaching a JSI of 17%. Studies from 1982 and 1998 had seven species in common (23%).

**Table 1:** Native bee species found in Farellones, Chile. Each species identified in the three published works is indicated with an “X”.

No	**Family** species	Survey 2016–2018^*^	Camousseight & Barrera (1998)	Arroyo, Primack & Armesto (1982)
	**Andrenidae**			
1	*Acamptopoeum submetallicum* Spinola^**^	X		
2	*Euherbstia excellens* Friese^**^	X		
3	*Liphanthus andinus* Ruz & Toro^**^	X		
4	*L. coquimbensis* Ruz & Toro^**^	X		
5	*L. sabulosus* Reed	X		X
6	*Rhophitulus evansi* Ruz & Chiappa^**^	X		
	**Colletidae**			
7	*Cadeguala occidentalis* Haliday	X		X
8	*Caupolicana bicolor* Friese^**^	X		
9	*C. dimidiata* Herbst^a^			X
10	*Chilicola curvapeligrosa* Monckton^**^	X		
11	*Colletes kuhlmanni* Ferrari^**^	X		
12	*C. fulvipes* Spinola	X	X	X
13	*C. musculus* Friese^**^	X		
14	*C. sulcatus* Vachal^a^		X	X
15	*Hemicotelles ruizii* Herbst^a^			X
16	*Xanthocotelles incahuasi* Toro & Cabezas^a^			X
17	*X. sicheli* Vachal^a^			X
18	*Xeromelissa farellones* Toro & Moldenke^**^	X		
19	*Xeromelissa sororitatis* Henríquez-Piskulich, Villagra & Vera, n. sp. ^b^^**^	X		
	**Halictidae**			
20	*Caenohalictus aplacodes* Rojas & Toro^**^	X		
21	*C. iodurus* Vachal	X	X	
22	*C. rostraticeps* Friese^**^	X		
23	*Callistochlora chloris* Spinola^**^	X		
24	*C. prothysteres* Vachal^**^	X		
25	*Ruizanthedella cerdai* Rojas^**^	X		
26	*R. mutabilis* Spinola	X		X
27	*R. nigrocaerulea* Spinola^**^	X		
28	*Ruizantheda proxima* Spinola^**^	X		
29	*Sphecodes granulosus* Sichel^**^	X		
	**Megachilidae**			
30	*Anthidium chilense* Spinola	X		X
31	*A. chubuti* Cockerell^**^	X		
32	*A. decaspilum* Moure^a^		X	X
33	*A. espinosai* Ruiz	X		X
34	*A. funereum* Schletterer^a^			X
35	*A. gayi* Spinola^a^			X
36	*A. rubripes* Friese^a^			X
37	*Megachile distinguenda* Ruiz^**^	X		
38	*M. pollinosa* Spinola	X	X	
39	*M. saulcyi* Guérin-Méneville	X		X
40	*M. semirufa* Sichel	X	X	X
41	*Trichothurgus herbsti* Friese^**^	X		
42	*T. wagenknechti* Moure^a^			X
	**Apidae**			
43	*Alloscirtetica gayi* Spinola	X		X
44	*A. rufitarsis* Bertoni	X	X	X
45	*Anthophora incerta* Spinola	X		X
46	*Apis mellifera* Linnaeus	X		
47	*Bombus dahlbomii* Guérin-Méneville	X		X
48	*Bombus terrestris* Linnaeus	X		
49	*Centris cineraria* Smith	X	X	X
50	*C. nigerrima* Spinola	X	X	X
51	*Chalepogenus caeruleus* Friese	X		X
52	*C. herbsti* Friese		X	X
53	*Diadasia chilensis* Spinola^**^	X		
54	*Epiclopus gayi* Spinola	X		X
55	*E. lendlianum* Friese	X	X	
56	*Isepeolus luctuosus* Spinola^**^	X		
57	*Melectoides niveiventris* Friese^a^			X
58	*Svastrides melanura* Spinola	X		X
**Number of shared species with currentsurvey**			8	17

**Notes.**

* January 2019 was also sampled for this survey.

** Species added to the previous published lists of bee species for this area.

a Species not found in the present work but listed for this area in previous works.

b Described as *Xeromelissa* sp. in Henríquez-Piskulich et al. (2018).

**Figure 1:**
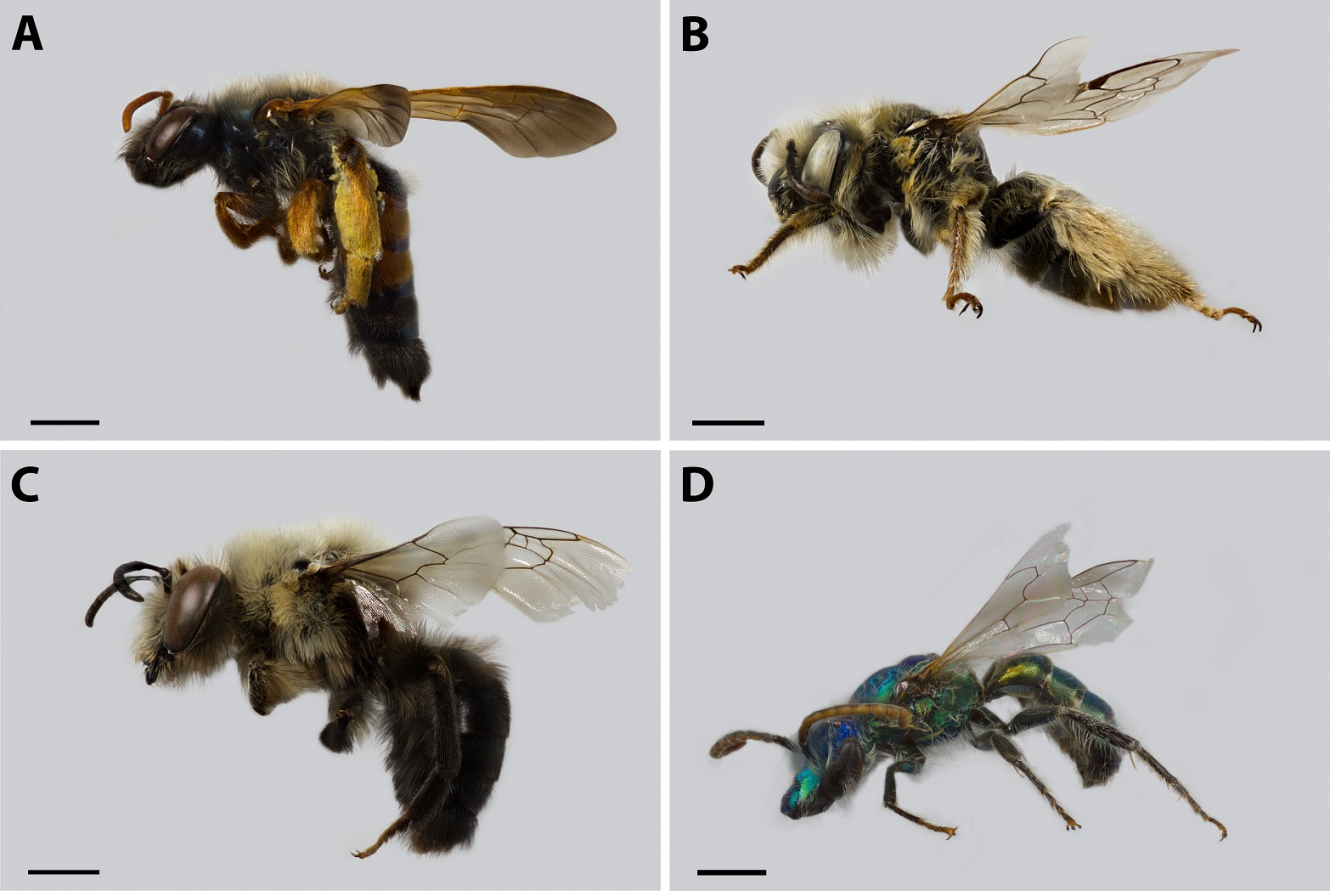
Some of the specimens reported for the first time for this area of study. ****(A) *Euherbstia excellens* (Andrenidae), scale bar 2.5 mm; (B) *Diadasia chilensis* (Apidae), scale bar 1.5 mm; (C) *Caupolicana bicolor* (Colletidae), scale bar 2.5 mm; (D) *Caenohalictus rostraticeps* (Halictidae), scale bar 1.5 mm.

In this work, 26 species were collected for the first time in this area of study, adding to the previous published lists of species for this location: *Acamptopoeum submetallicum* Spinola*, Euherbstia excellens* Friese*, Liphanthus andinus* Ruz & Toro*, L. coquimbensis* Ruz & Toro*, Rhophitulus evansi* Ruz & Chiappa*, Caupolicana bicolor* Friese*, Chilicola curvapeligrosa* Monckton*, Colletes kuhlmanni* Ferrari*, C. musculus* Friese*, Xeromelissa farellones, X. sororitatis,* **n. sp.**, *Caenohalictus aplacodes* Rojas & Toro*, C. rostraticeps* Friese*, Callistochlora chloris* Spinola*, C. prothysteres* Vachal*, Ruizanthedella cerdai* Rojas*, R. nigrocaerulea* Spinola*, Ruizantheda proxima* Spinola*, Sphecodes granulosus* Sichel*, Anthidium chubuti* Cockerell*, Megachile distinguenda* Ruiz*, Trichothurgus herbsti* Friese*, Apis mellifera* Linnaeus, *Bombus terrestris* Linnaeus, *Diadasia chilensis* Spinola and *Isepeolus luctuosus* Spinola (Fig. 1).

There were 12 species which were not found in the present work, but were listed for this area: *Caupolicana dimidiata* Herbst*, Colletes sulcatus* Vachal*, Hemicotelles ruizii* Herbst*, Xanthocotelles incahuasi* Toro & Cabezas*, X. sicheli* Vachal*, Anthidium decaspilum* Moure*, A. funereum* Schletterer*, A. gayi* Spinola*, A. rubripes* Friese*, Trichothurgus wagenknechti* Moure*, Chalepogenus herbsti* Friese and *Melectoides niveiventris* Friese. From this list, only *C. sulcatus, A. decaspilum* and *C. herbsti* were reported in Camousseight & Barrera (1998) and Arroyo, Primack & Armesto (1982); all other species were only found in the latter study.

### Bee family abundance and phenology

Specimens were collected in different amounts during 2017–2018 field survey (Chi square, *n* = 840, *df* = 4, *X*^2^ = 488.82, *p* < 0.0001). Halictidae corresponded to nearly half of the total bees collected (48.8%) differing with statistical significance from the other groups. It was followed by Apidae (20.2%), Andrenidae (15.6%), Colletidae (10%) and Megachilidae (5.4%) (See Table S2). Differences were also found at genus level (Chi square, *n* = 825, *df* = 16, *X*^2^ = 1261.1, *p* < 0.0001), with *Lasioglossum* corresponding to 30% of specimens and *Bombus* (represented only by native species *B. dahlbomii*) 14% of specimens collected by the recent survey (See Table S3 and the supporting dataset for abundance results).

**Figure 2:**
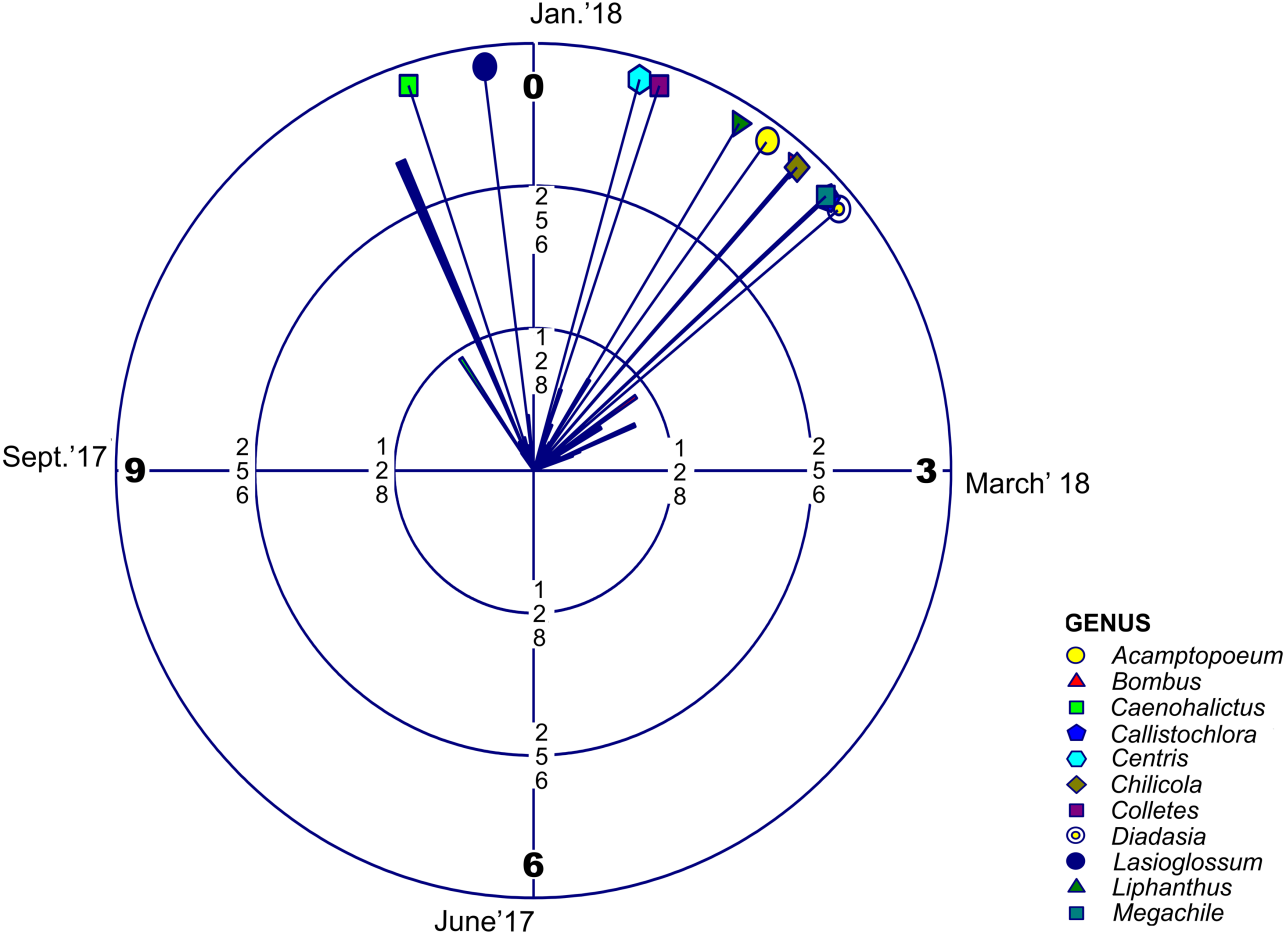
Circular Histogram Rose Plot. Representing a year clock which corresponds to the field season 2017–2018. Different colors and geometric shapes represent different bee genera. Arrows with different colors show the mean time of emergence of each group.

Regarding adult phenology, we found that four of these families concentrated their occurrences in January (month 1) and only Halictidae differed with a main peak near December (Fig. 2, Table 2 and Table 3). Moreover, mean resultant length (R bar value) was near one for the five bee families. This accounted for an elevated temporal concentration of the collected adult bees from all these groups, accompanied by a concomitant reduced circular variance (v) (Table 2). When evaluating uniform distribution goodness-of-fit for temporal occurrence of adult bees from each family, using Rayleigh (z) test, it was possible to find that the concentration and direction of the main peak of adult collection found in each family was statistically significant (Table 3). Furthermore, multivariate circular comparison shows that, in general, these families differed in the distribution of adult collections (Mardia-Watson-Wheeler Uniform Scores Test; *W*: 254.295; *p* < 0.001). Post hoc two groups test showed that despite the finding that most bee families concentrated in the summer season, they differed with statistical significance in their temporal distribution (Table S4). Only when comparing Colletidae vs. Megachilidae or vs. Apidae their distribution were considerably overlapped. Also, Apidae adult bees were collected mainly around January (Table S4).

**Table 2:** Summary of circular statistics for native bee genera. Family and genus are shown in the first two columns, followed by their sample sizes (N) and the mean direction of data dispersion through the year clock (Theta). Theta corresponds to the time of year when bees were most abundant on average. Data concentration is shown with R bar values, where the R bar near 1 implies a high concentration of values, Circular Standard Deviation (v) is shown in the following column, with values near zero representing elevated concentration of collections. This is followed by Circular Dispersion (Delta) values for each genus, corresponding to the circular dispersion of data, and the kurtosis (Von Mises distribution Kappa values). If the kurtosis is greater than zero, then the distribution has heavier tails. Last column corresponds to the results of the S statistics for Rayleigh (z) test uniform distribution goodness-of-fit. Asterisks highlight significant values at significance *p* < 0.05.

		**Sample**	**Mean**	**Mean resultant**	**Circular**	**Circular**	**Von Mises**	**Rayleigh’s**	
		**Size**	**Direction**	**Length**	**SD**	**Dispersion**	**Concentration**	**Test Statistic**	
**Family**	**Genus**	**(N)**	**(Theta)**	**(R bar)**	**(v)**	**(Delta)**	**(Kappa)**	(S*)	
Andrenidae	*Acamptopoeum*	20	1.20	0.97	0.51	0.071	14.55	44.99	*
Apidae	*Bombus*	113	1.38	0.90	0.88	0.192	5.23	218.40	*
Halictidae	*Caenohalictus*	88	11.39	0.92	0.78	0.141	6.59	179.95	*
Halictidae	*Callistochlora*	62	1.59	0.96	0.58	0.085	11.55	138.20	*
Apidae	*Centris*	23	0.52	0.95	0.58	0.094	11.31	50.57	*
Colletidae	*Chilicola*	29	1.39	0.94	0.67	0.125	8.72	61.807	*
Colletidae	*Colletes*	44	0.62	0.80	1.29	0.541	2.83	64.11	*
Apidae	*Diadasia*	15	1.67	0.97	0.47	0.063	13.60	33.91	*
Halictidae	*Lasioglossum*	252	11.76	0.81	1.24	0.412	2.99	383.47	*
Andrenidae	*Liphanthus*	88	1.04	0.90	0.85	0.19	5.56	172.81	*
Megachilidae	*Megachile*	33	1.58	0.96	0.55	0.084	12.45	73.73	*

### Taxonomy

**Table:** 

**Figure 3:**
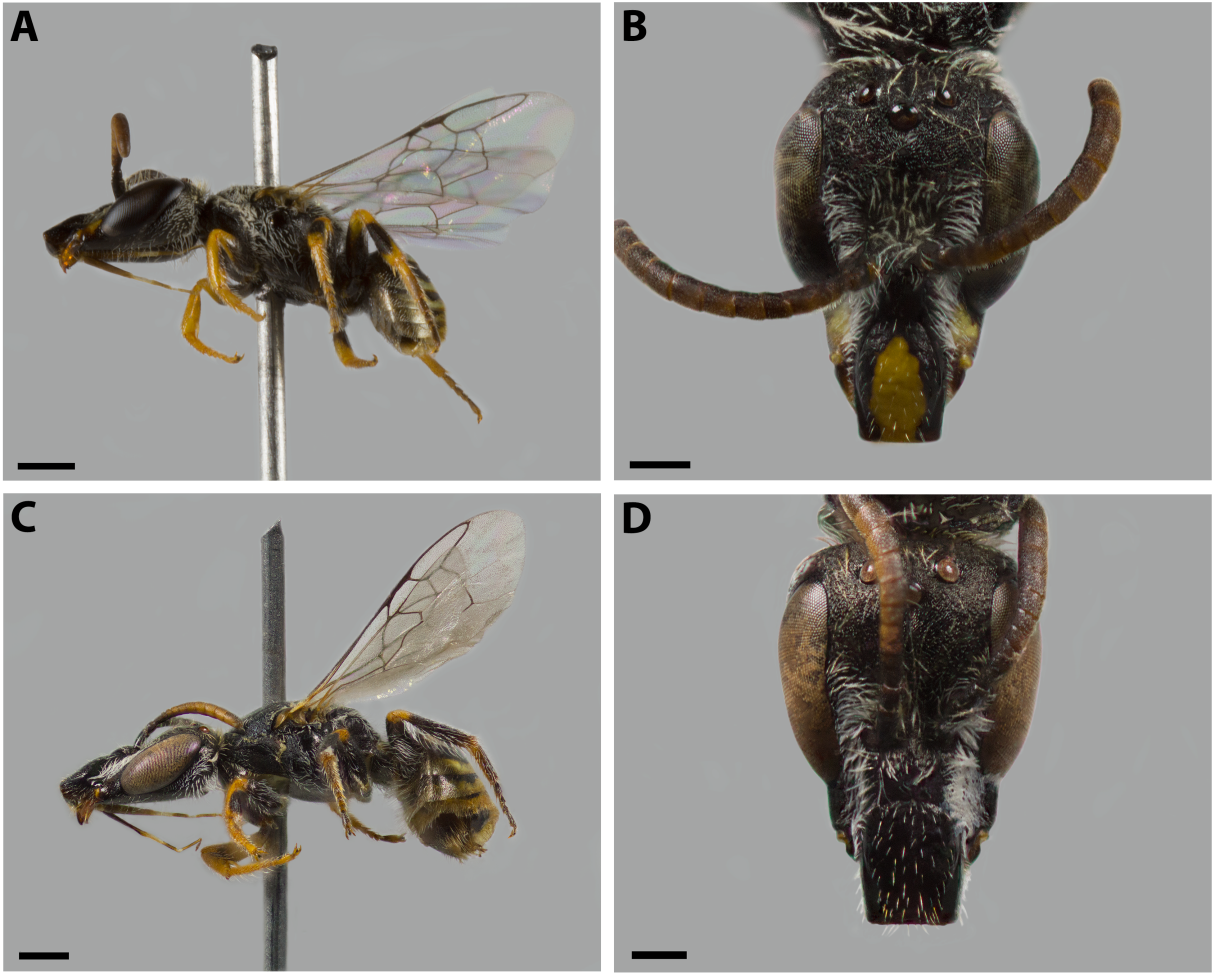
*Xeromelissa sororitatis* n. sp. Male: (A) habitus, lateral view, scale bar 0.5 mm; (B) face, frontal view, scale bar 0.25 mm. Female: (C) habitus, lateral view, scale bar 0.5 mm; (D) face, frontal view, scale bar 0.25 mm.

**Figure 4:**
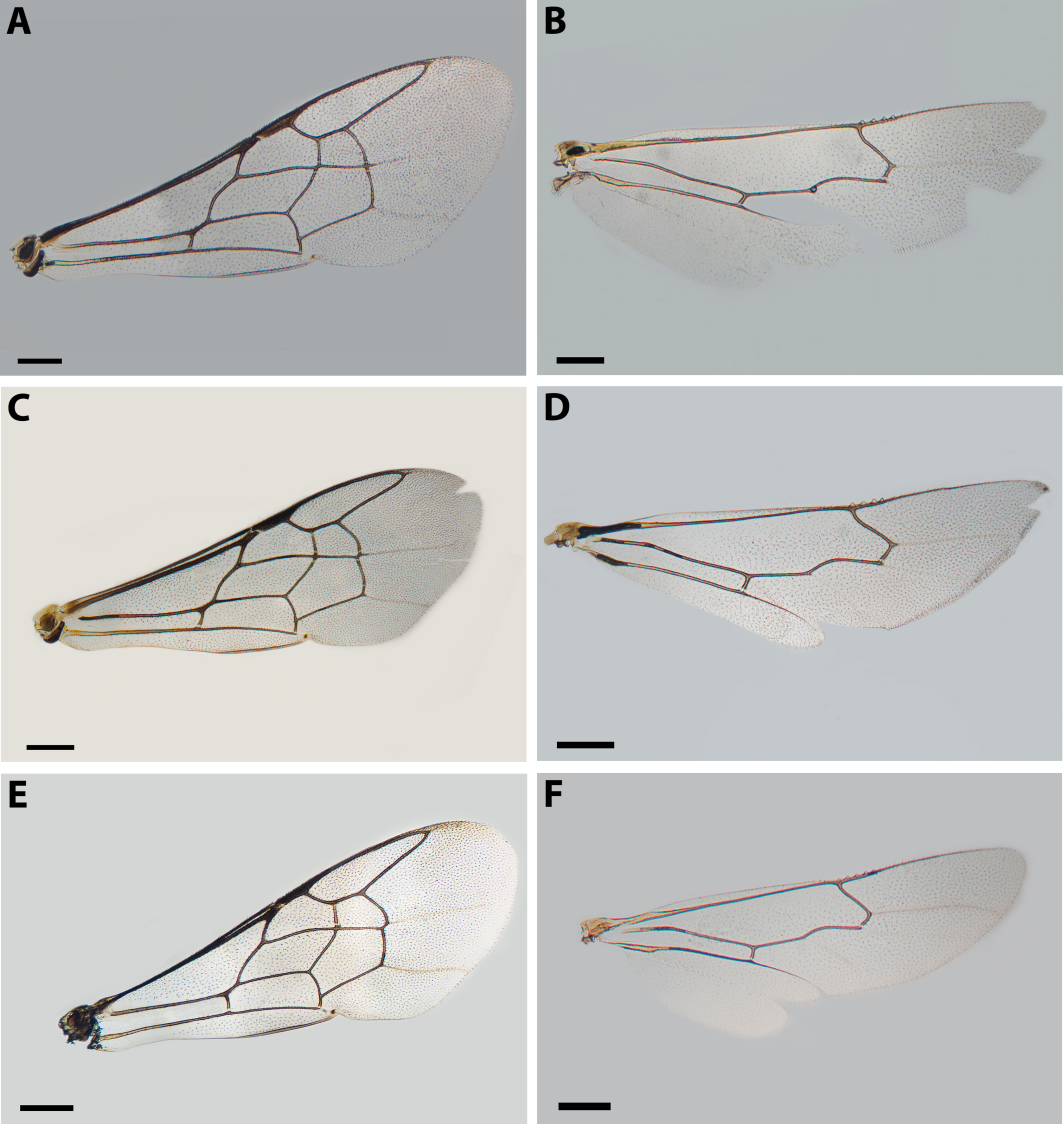
Wings of *X. sororitatis* n. sp. and *X. farellones*. (A–D) *X. sororitatis.* Male: (A) anterior wing, scale bar 0.225 mm; (B) posterior wing, scale bar 0.186 mm. Female: (C) anterior wing, scale bar 0. 312 mm; (D) posterior wing, scale bar 0.249 mm. (E–F) *X. farellones*. Female: (E) anterior wing, scale bar 0.288 mm; (F) posterior wing, scale bar 0.216 mm.

**Figure 5:**
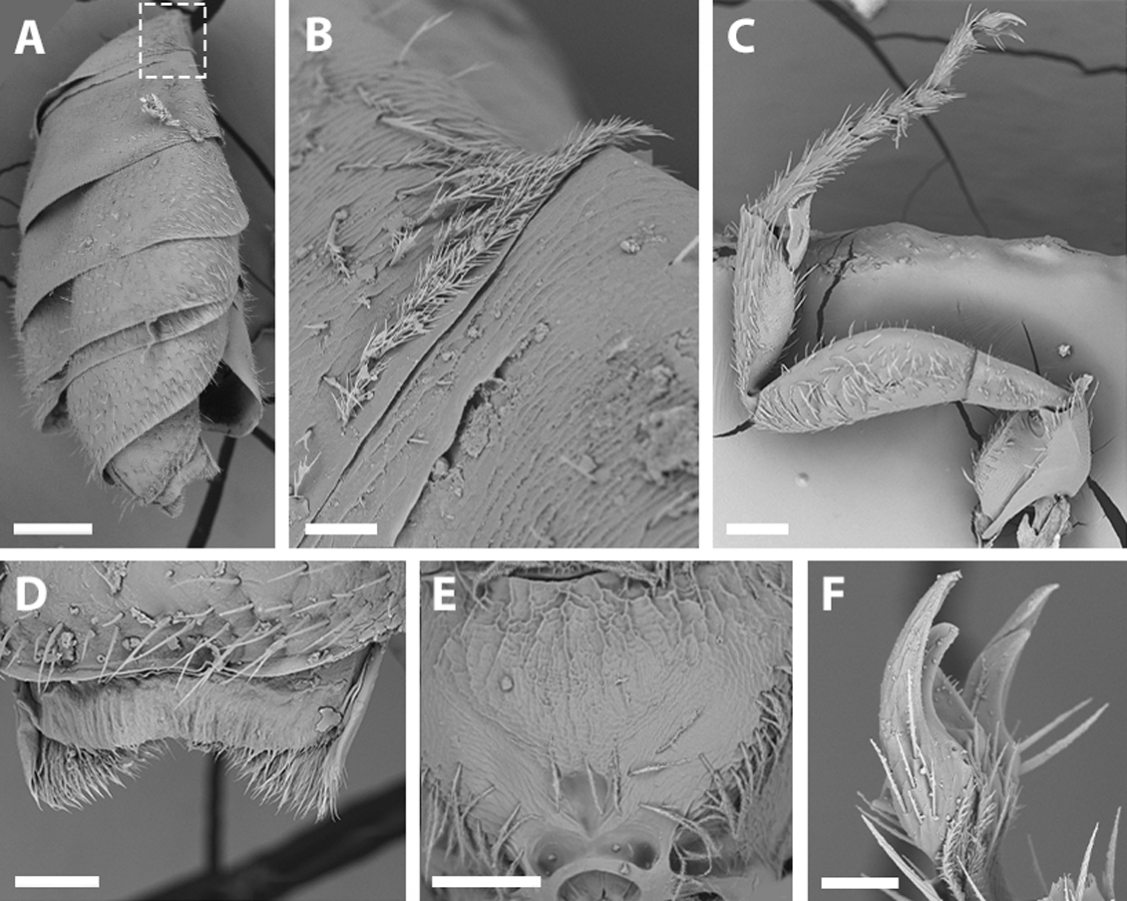
SEM of male *X. sororitatis.* n. sp. (A) Abdomen, scale bar 0.3 mm; (B) hairs on declivitous surface of T1, scale bar 30 µm; (C) anterior leg, scale bar 150 µm; (D) T7, scale bar 60 µm; (E) propodeum longitudinally striate, scale bar 150 µm; (F) claws of anterior leg, scale bar 30 µm.

**Figure 6:**
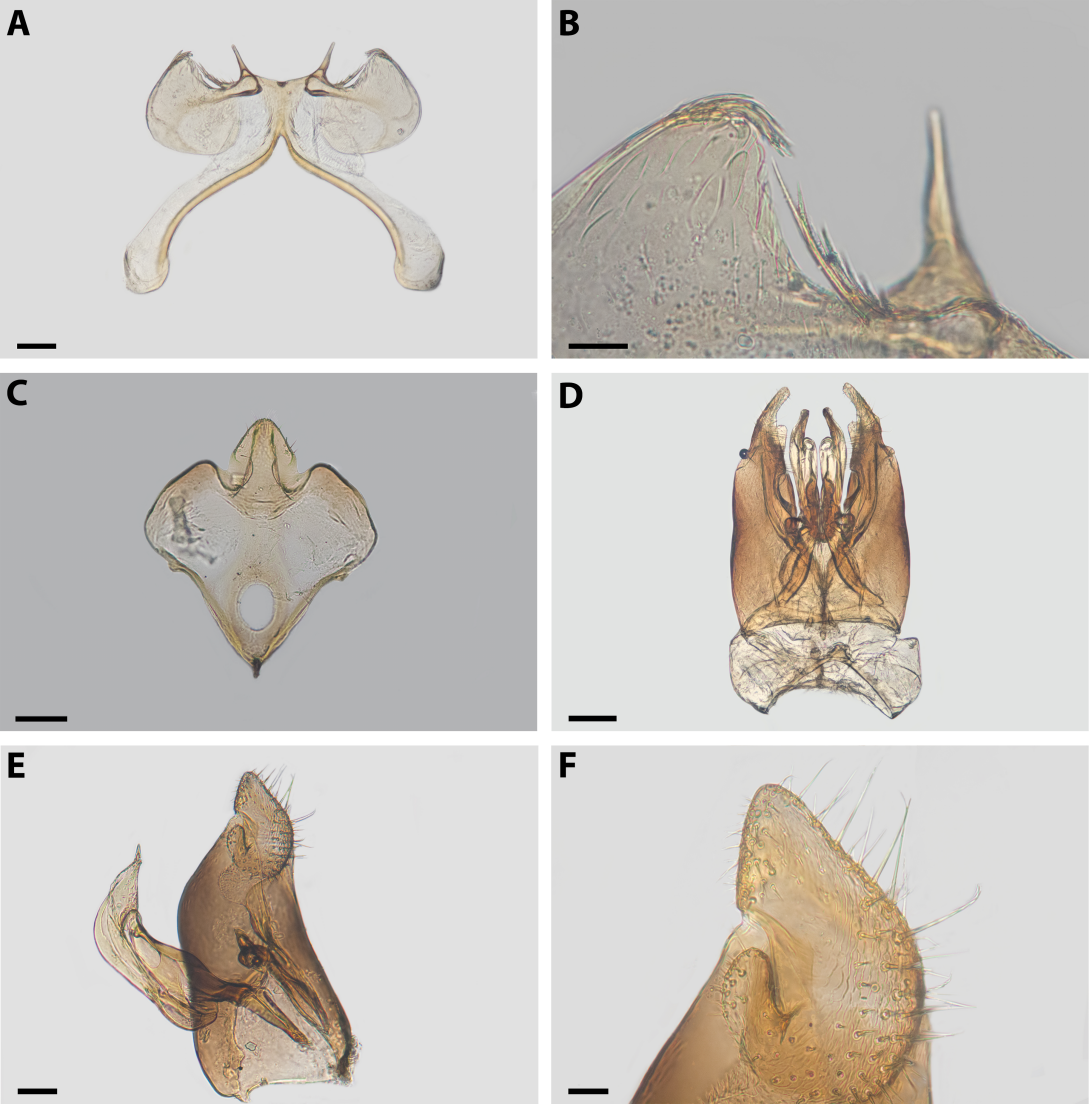
Male terminalia of *X. sororitatis.* n. sp. (A) S7, scale bar 0.09 mm; (B) anterior margin of S7, scale bar 0.024 mm; (C) S8, scale bar 0.096 mm; (D) dorsal view of genital capsule, scale bar 0.12 mm; (E) inner view of genital capsule, scale bar 0.036 mm; (F) inner view of gonostylus, scale bar 0.012 mm.

**Figure 7:**
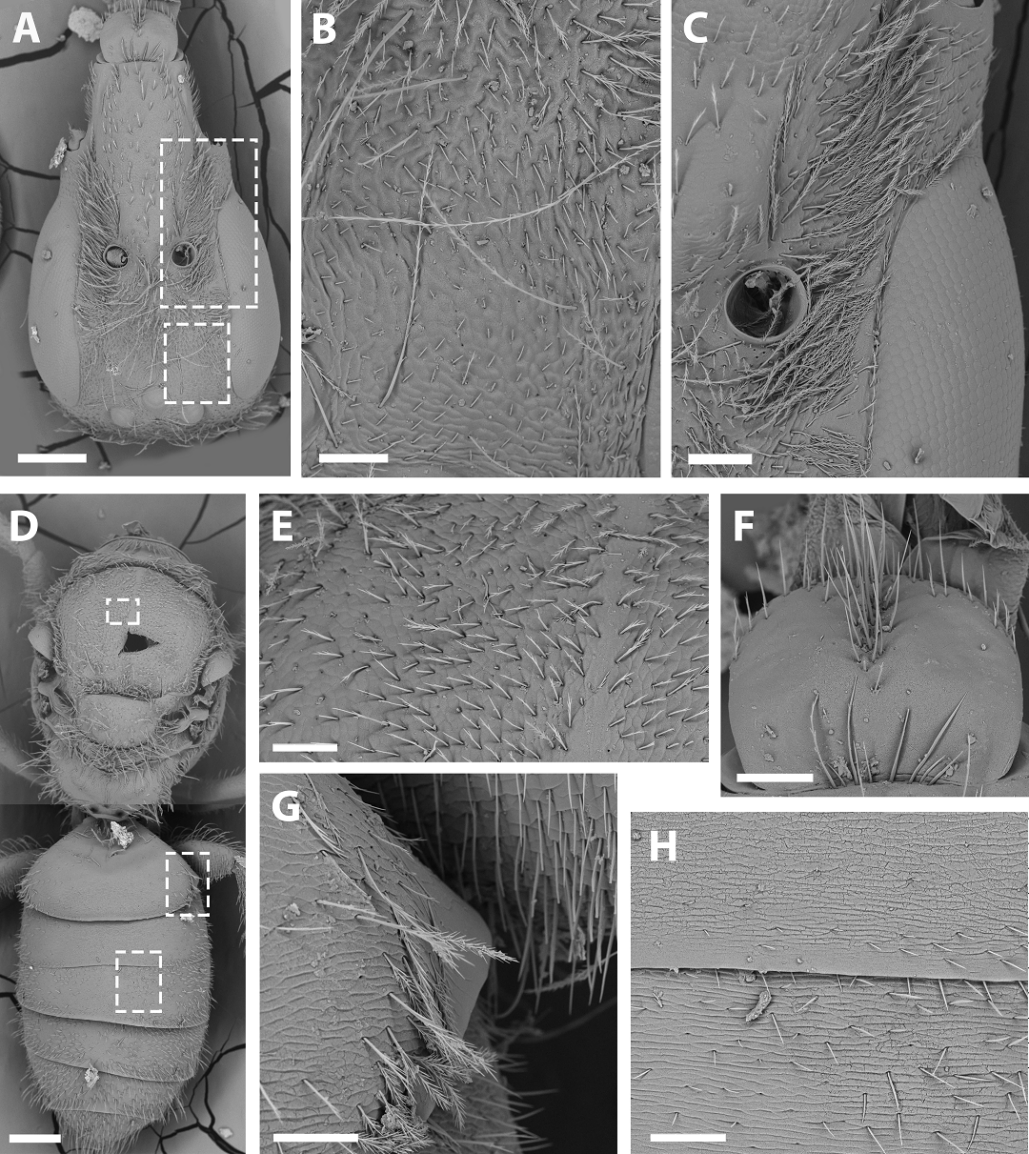
SEM of female *X. sororitatis* n. sp. (A) Head, scale bar 0.3 mm; (B) vertex, scale bar 60 µm; (C) paraocular area, scale bar 90 µm; (D) dorsal view of the body, scale bar 0.3 mm; (E) hairs of thorax, scale bar 60 µm; (F) hairs of labrum, scale bar 90 µm; (G) hairs on declivitous surface of T1, scale bar 60 µm; (H) transversal striae of metasoma, scale bar 60 µm.

**Figure 8:**
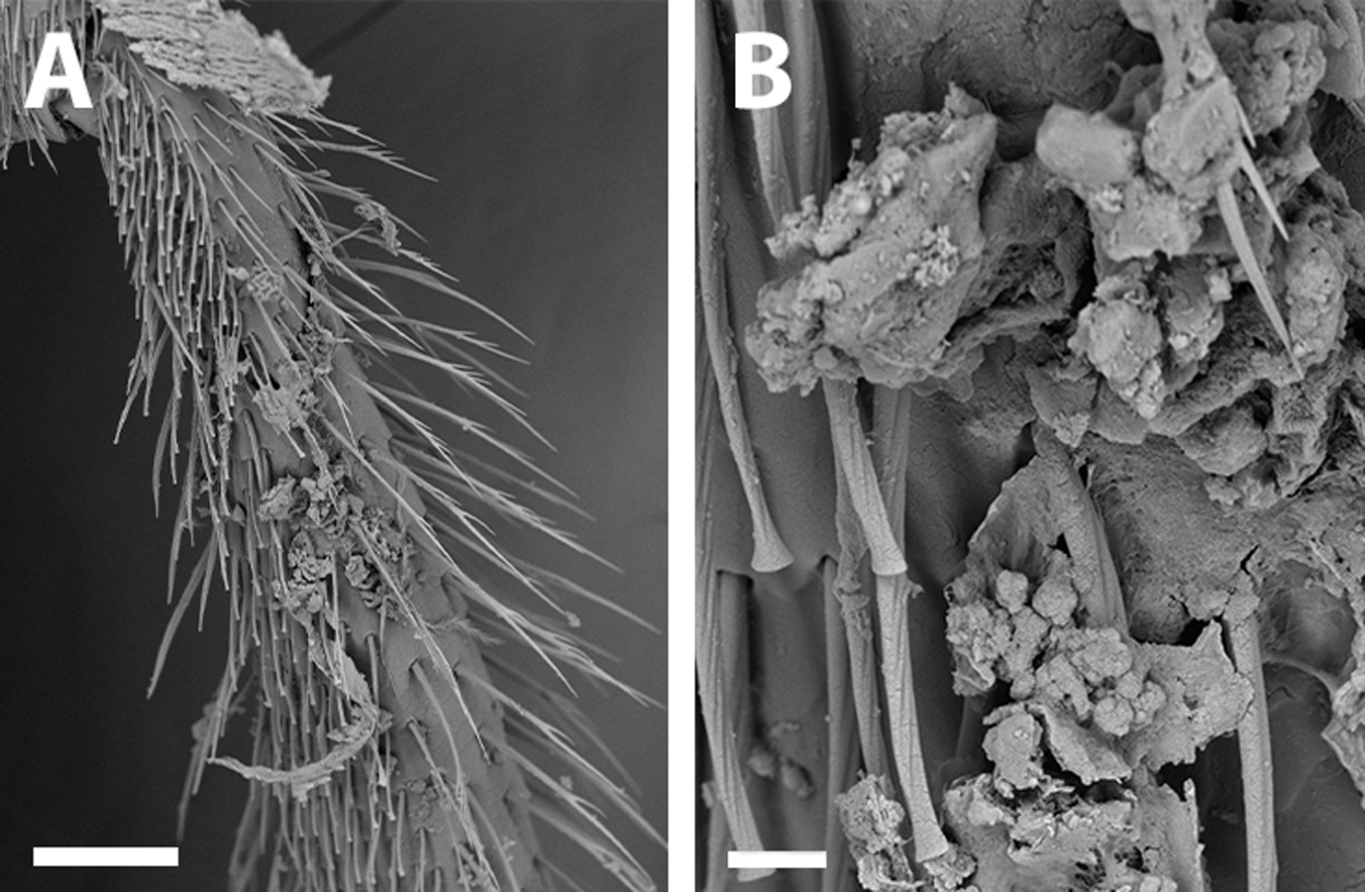
SEM of hind tibia of female *X. sororitatis* n. sp. (A) Hind tibia hairs, scale bar 90 µm; (B) wing grooming hairs, scale bar 9 µm.

*Xeromelissa sororitatis***Henríquez-Piskulich, Villagra & Vera new species**
(Figs. 3A–3D, 4A–4D, 5A–5F, 6A–6F, 7A–7H, 8A–8B)

**Diagnosis.** ***Xeromelissa sororitatis*** males can be differentiated from those of *X. farellones* by the absence of the inner spine on profemurs and the genitalia, especially the spine-like processes on the anterior margins of S7 and the absence of a long hairy process on the anterior margin of S8. The male terminalia of this new species is similar to that of *X. nortina* Toro & Moldenke but can be distinguished by the more robust anterior lobules of S7 (Fig. 6). The new species can also be distinguished from *X. nortina* by the upper interorbital distance being similar to the lower, the scape being more than 2x as long as its greatest width, and the labrum being two times broader than long. The female of *X. sororitatis* can be differentiated from that of *X. farellones* by the longer malar space (13:19), labrum with its anterior margin more rounded, denser pilosity and with hairs starting at its center that continue towards the anterior margin. The female of *X. nortina* has yet to be described.

**Description. Holotype male.** ***Dimensions:*** Body length: 3.6 mm. Head length: 1.5 mm. Head width: 1.1 mm. Fore wing length: 2.7 mm. ***Coloration*** (Figs. 3A–3B; 4A–4B)***:*** Black, with following parts yellow: malar space, clypeus, proximal 1/4 of mandible with a yellow spot, ventral surface of flagellum, tegula dark amber with a spot, wing veins towards base, terga I-VII with a stripe that presents slits on both sides of their anterior margin, last tergite, inner and distal halve of outer lateral profemur, protibia and protarsi; 1/4 of the femur, basal and distal part of the tibia, basitarsi of the second and third pair of legs (rest of the tarsi are dark amber). ***Pubescence:*** White, thick, dense, and plumose on paraocular, genal, pronotal and pleural areas, margins of scutum, scutellum, metanotum and declivitous surface of T1 (Figs. 5A–5B); white and thin with fewer or no branches on frons and occipital margin. ***Surface Sculpture*:** Integument surface shiny, with a uniform and very shallow microsculpture. Frontal area, paraocular area and vertexal area with fine and deep striae (Figs. 7B–7C, same as in the female). Propodeum longitudinally striate in the anterior margin and slightly wrinkled transversally (Fig. 5E). Metasoma with fine, shallow and uniform transverse striae (Fig. 7H, same as in the female).

**Table 3:** Rayleigh (z) test uniform distribution goodness-of-fit. This table shows bee Family, sample sizes (N), followed by S statistical values for each group accompanied by significance at *p* < 0.05. Asterisks highlight significant values.

**Family**	**N**	**S**	***p***	
Andrenidae	131	246.20	0.0	*
Colletidae	84	140.98	0.0	*
Halictidae	410	598.86	0.0	*
Megachilidae	45	101.21	0.0	*
Apidae	170	325.58	0.0	*

***Structure:*** *Head:* Same length as mesosoma (1:1) and 1.5x as long as wide (3:2). Upper interorbital distance similar to the lower, and shorter than eye length (7:7:8). Compound eye with inner margin weakly concave (Fig. 3B; 7A, same as in the female). Clypeus convex and projected below the orbital tangent, longer than its greatest breadth (5:3); with a straight lower margin. Labrum two times broader than long, apex rounded. Mandible with subapical tooth small and rounded. Malar space as long as the basal depth of mandible, with malar line weakly visible. Subantennal sutures clearly noticeable. Paraocular area medially weakly depressed towards alveolus. Alveolus diameter similar to the distance between compound eye and alveolus. Supraclypeal area convex in lateral view. Genal area a third of compound eye breadth (9:28), in profile the margin of the latter parallel with that of the genal area ventrally. Scape more than 2x as long as its greatest width (13:6). F1 slightly longer than pedicel and F2 (11:10:8). Length of first and sixth maxillary palpi longer than the rest (9:5:8:3:4:9).

*Mesosoma:* Pronotum with defined collar. Ratio of scutellum:metanotum:horizontal surface of metapostnotum lengths 25:14:15. Episternal groove noticeable. Profemur without basal inner spur (Fig. 5C). Metatrochanter without basal projection; metafemur broad and metatibia broadened basally, with long and narrow spurs. Bifurcated claws (Figs. 5C, 5F). Fore wings with first submarginal cell almost two times the length of the second (8:5) (Fig. 4A).

**Figure 9:**
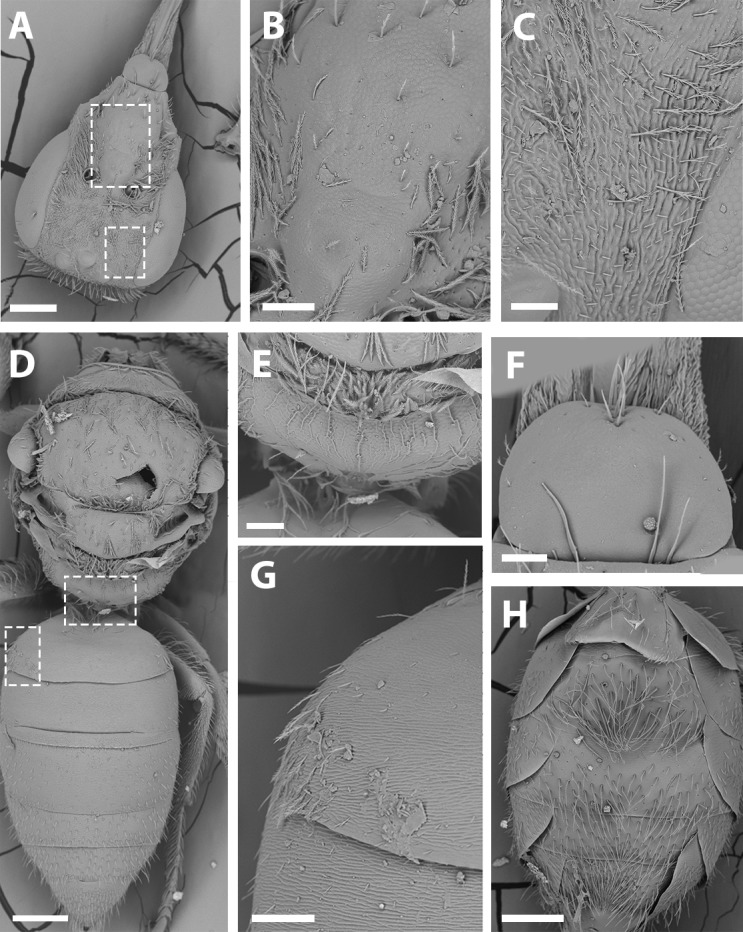
SEM of female *X. farellones*. (A) Head, scale bar 0.3 mm; (B) frons, scale bar 90 µm; (C) paraocular area, scale bar 60 µm; (D) dorsal view of the body, scale bar 0.3 mm; (E) propodeum, scale bar 90 µm; (F) hairs of labrum, scale bar 60 µm; (G) hairs on declivitous surface of T1, scale bar 90 µm; (H) ventral view of metasoma, 0.3 mm.

*Metasoma:* Convex, broadest at T3. Posterior margin of T7 bifurcated with posterior margin hirsute (Fig. 5D). Terminalia: As in Fig. 6.

**Dimensions of the Paratype:** Body length: 3.7 mm. Head length: 1.5 mm. Head width: one mm. Fore wing length: 2.8 mm.

**Female.** ***Average Dimensions*** Paratype (Range): Bigger than male. Body length: 5.1 mm (5.1–5.3 mm). Head length: 1.7 mm (1.6–1.7 mm). Head width: 1.1 mm (1.1–1.2 mm). Fore wing length: 3.1 mm (3.1–3.3 mm). ***Coloration*** (Figs. 3C–3D; Figs. 4C–4D): Black, with following parts yellow: proximal 1/4 of mandible with a yellow spot and following 2/4 varying from yellow to amber, in some cases clypeus with small medial patch, ventral surface of flagellum, tegula dark amber, wing veins towards base, terga I-VII with a stripe that presents slits on both sides of their anterior margin, distal ring on profemur and the protibia (except for dark amber of outer lateral surface), femorotibial joint for the second and third pair of legs. Protarsi, mesotarsi and metatarsi except for dark amber outer lateral surface. ***Pubescence:*** Labrum with hairs starting at its center that continue towards its anterior margin (Fig. 7F); White, thick, dense, and plumose on paraocular, genal, pronotal and pleural areas, margins of scutum, scutellum, metanotum and declivitous surface of T1 (Fig. 7G); white and thin with fewer or no branches on frons and occipital margin; metatibia with long hairs with few branches on scopa laterally and long wing grooming hairs on inner medial surface (Figs. 8A–8B). Long hairs in sternites II-IV. ***Surface Sculpture:*** Integument surface shiny, with a uniform and very shallow microsculpture. Frontal area, paraocular area and vertexal area with fine and deep striae (Figs. 7B–7C). Propodeum longitudinally striate in the anterior margin and slightly wrinkled transversally (Fig. 5E, same as in the male). Metasoma with fine, shallow and uniform transverse striae (Fig. 7H). ***Structure:*** *Head:* Same length as mesosoma (1:1) and 1.6x as long as wide (17:11). Apical interorbital distance similar to the inferior, and shorter than eye length (14:13:18). Compound eye with inner margin weakly concave (Figs. 3D; 7A). Clypeus convex and projected below the orbital tangent, longer than its greatest breadth (17:13); with a straight distal margin. Labrum almost two times broader than long (11:7), apex rounded (Fig. 7F). Mandible with subapical tooth small and rounded. Malar space slightly longer than the basal depth of mandible (19:9), with malar line weakly visible as in male. Subantennal sutures clearly noticeable. Paraocular area medially weakly depressed towards alveolus. Alveolus diameter similar to the distance between compound eye and alveolus. Supraclypeal area convex in lateral view. Genal area a little less than half compound eye breadth (17:43), in profile the margin of the latter parallel with that of the genal area ventrally. Scape more than two times as long as greatest width (5:2). F1 slightly shorter than pedicel and similar to F2 (9:11:6). Length of sixth maxillary palp longer than the rest (7:4:7:4:3:9).

*Mesosoma* (Figs. 7D–7E): Pronotum as in male. Ratio of lengths scutellum:metanotum:horizontal surface of metapostnotum 12:7:6. Episternal groove noticeable. Legs without modifications. Bifurcated claws. Fore wings with first submarginal cell a little less than two times the length of the second (7:4).

*Metasoma* (Figs. 7D, 7G, 7H): Convex, broadest at T3.

**Material Studied.** Male holotype: CHILE: *REGIÓN METROPOLITANA:* Santiago, Farellones, S 33°22.084′, W 070°16.264′, 2,465 m; pan traps, 2.xii.2017, P. Henríquez-Piskulich; five paratypes: one female with same data as the holotype; one male and three females with same data as the holotype but from 2.ii.2018. Holotype and paratypes are deposited at the entomological collection of the National Museum of Natural History, Santiago-Chile (MNHNC).

**Etymology.** Sororitas: sisterhood; tis: genitive singular of third declension. The specific epithet is inspired by and honors all hardworking women in STEM, in recognition of all the difficulties they must face every day and the importance of protecting each other as sisters would. It is to be treated as a noun.

**Remarks:** SEM photographs show that the hind tibiae of both *X. sororitatis* and *X. farellones* have long hairs with few branches on outer lateral surface of scopae and long wing grooming hairs on inner medial surface (Figs. 8A–8B). Also, with SEM it can be seen that the integument of both species is not punctate as previously described by Toro & Moldenke (1979), but rather shiny, with a uniform and very shallow microsculpture, where in some cases a fine and deep striae is present (Figs. 7B–7C, same as in the female; Figs. 9B–9C). We also show that the forewings of *X. sororitatis* may have four to five hamuli, whereas those of *X. farellones* always have four (Figs. 4B, 4D, 4F). The labrum of females of each species is different in shape and pubescence, and could potentially be used as a way to differentiate between females of this genus (Figs. 7F; 9F).

## Discussion

The geographical area surveyed in this work is part of the Maipo river basin, which extends between the latitudes 32°S to 34°S, and the longitudes 69°W and 71°W (Niemeyer, 2015). Between latitude points in this basin, there are approximately 266 bee species (Montalva & Ruz, 2010), 54 of which are represented in the high-altitude area surveyed in this work (20.3%). Considering the present list has a total of 58 species, there are four species which have not been reported to be between these two latitude points: *Liphanthus andinus*, *Xanthocotelles incahuasi*, *X. sicheli* and *Xeromelissa sororitatis*, **n. sp.** Specimens identified as belonging to these species have only been reported for *L. andinus* (Montalva & Ruz, 2010), and *X. sororitatis* which is a new species described in the present work. The remaining two were recorded by Arroyo and colleagues (1982), and the material could not be examined. Additionally, this area is also part of a biodiversity hotspot called “Chilean winter rainfall–Valdivian forests”, which spans from the Pacific Coast to the crest of the Andean mountains between 25°S and 47°S (Arroyo et al., 2004). This hotspot has approximately 337 native bee species that have been recorded (Montalva & Ruz, 2010), where this area of study presented around 16.6% of this total. Considering mountains could function as reservoirs of biodiversity, supplying other ecosystems with species which possibly buffer insect declines (Sedano & Burns, 2010), it is crucial to create policies that protect high-altitude ecosystems.

The present work in Farellones added 26 bee species to the previous published lists of species for this area, including among them a new species of *Xeromelissa*. To the best of our knowledge, eight of these species have been previously found in the same area: *R. evansi, C. curvapeligrosa, C. kuhlmanni, C. musculus, X. farellones, C. aplacodes, C. rostraticeps,* and *A. chubuti* (Toro & Moldenke, 1979; Toro & Rodríguez, 1998; Ruz & Chiappa, 2004; Monckton, 2016; Ferrari, 2017). The other 18 species are *A. submetallicum, E. excellens, L. andinus, L. coquimbensis, C. bicolor, X. sororitatis*, **n. sp.**, *C. chloris, C. prothysteres, R. cerdai, R. nigrocaerulea, R. proxima, S. rugulosus, M. distinguenda, T. herbsti, A. mellifera*, *B. terrestris*, *D. chilensis* and *I. luctuosus*.

Jaccard similarity index comparisons showed that the present work differed to a great extent to the two previous works. Heterogeneous floral and bee phenology could have an influence over insect sampling because of the mismatch between bee emergence, the flowers from which they forage, and sampling time (Kudo & Ida, 2013; González et al., 2016). This could be one of the reasons for the difference between the list of bee species in this work and former studies in the area. In the work of Arroyo and collegues (1982), the entire flowering season between October 1980 and March 1981 was sampled. The present survey however, did not sample the months of October and March, and as a result, it could be possible that some of the previously recorded species weren’t found because of a mismatch between bee phenology and sampling period. Despite these temporal differences, the recent collection was able to record more species. This could be due to an inadequate sampling method for covering a significant portion of bee biodiversity (McCravy & Ruholl, 2017), given that the survey published in 1982 mostly considered insects visiting flowering plants and had in their collection protocol a brief 10 min observation period at morning, midday and afternoon. The recent sampling may be more representative for this area because it considered a longer active collection with passive pan trapping. In contrast, the work of Camousseight & Barrera (1998), which shared 17% of similarity with the recent survey, only sampled 3 days during the month of January for the years 1979, 1980 and 1981. This could be why only eleven bee species were collected. Such brief windows of collection may only cover a small portion of the native bee assemblage found in this location (Fig. 2). This illustrates the importance of sampling for long periods of time in order to have a complete knowledge of the species that are part of an ecosystem. In addition, elevation may play an important role in differentiating these studies, given that higher elevations have reduced insect abundance and biodiversity (McCoy, 1990; Hodkinson, 2005). The elevation at the site selected for the former study by Camousseight & Barrera (1998) was 3,000 masl., whereas the present survey averaged 2,360 masl.

Concerning the bee species reported by previous lists not found in the current survey, *Colletes araucariae* Friese, a new synonym of *C. sulcatus* (Ferrari, 2017), was also present in the more recent work of Henríquez-Piskulich et al. (2018). However, with the re-examination of the material collected for this study and the work of Ferrari (2017), the collected specimens were correctly identified as *C. kuhlmanni.* Thus, it is impossible to rule out misidentifications as sources of discrepancies among the different studies conducted in the same area. Another possible reason this study didn’t find these species could be that the sites were not sampled frequently enough. If flowering periods of some plants are shorter than a month, and the associated bee species are only active in that time frame, they could potentially be missed during the sampling dates. In regards to the other species shared between these lists, there is a published record for *C. herbsti* (Roig-Alsina, 1999) but not for *A. decaspilum*. The remaining species not found in the current survey are all from Arroyo, Primack & Armesto (1982), but it was difficult to determine if they actually reside in this area because for this study it was not always deemed appropriate to collect the bees, but rather to identify them in the field. Methods such as this make it very difficult to validate taxonomic identifications because they hamper any possibility of verification (Packer et al., 2018). These false positives could cause bias in estimators of occupancy, colonization, and extinction (Miller et al., 2013), and could affect conservation measures regarding species that could be threatened (Richardson & Whittaker, 2010). These results highlight the lack of information that still exists regarding insect biodiversity, despite bees corresponding to a taxonomic group that gathers significant public attention among terrestrial invertebrates (Wilson, Forister & Carril, 2017).

Regarding abundance and phenology, halictids proved to be the most abundant bee family for this high-altitude area. Their phenological peak was around the end of November and December (Table 2), while the remaining four families were mainly found in January. Apidae was the second most abundant family, and the giant bumblebee *Bombus dahlbomii*, considered an endangered species by the IUCN (Morales et al., 2016), the most abundant species for this group. Mountain extreme habitats can work as high-altitude refuges (Tucker & Rehan, 2017), therefore if the subandean vegetational belt of Farellones were to be protected it may also be suitable for the conservation of this bumblebee.

For most genera of native bees, the occurrence of adults was temporally separated (Table 2). As in the case of some plants and their sequential flowering strategy, this allochrony could allow for reduced foraging competition between species (Stiles, 1975; Da Cruz, Mello & Van Sluys, 2006). Flowering phenology could be responsible for this difference in occurrence and if it is the case that there is temporal variation of floral resources, it would most likely affect bees (Ogilvie & Forrest, 2017). Temperature is a driver of plant phenology (Hodges, 1990; Tang et al., 2016), bee nesting and emergence periods (Nürnberger, Härtel & Steffan-Dewenter, 2018), and affects species distribution by removing or relocating barriers that allow species to change the ranges where they are present (Robinet & Roques, 2010). If temperature rises, flowering time might be reduced (Theobald, Breckheimer & HilleRisLambers, 2017), and could affect the emergence and temporal occurrence of these insects. This dyssynchrony between emergence of adult bees and the flowering period of their host plants has been suggested to be a potential scenario resulting from man-made environmental modifications such as erosion, habitat loss, urbanization and global warming patterns (Gottfried et al., 1999; Öztürk et al., 2015; Melo et al., 2017; Sánchez-Bayoa & Wyckhuys, 2019). Climate change could be outpacing phenological change for all plant species, and combined with early snowmelt could be playing a role in flowering phenology by reducing the probability of flowering (Wadgymar et al., 2018). Consequently, this could mean that previously recorded species for this area that didn’t appear in the recent survey are no longer present because they depended on a set of plants during a specific timeframe (Doi, Gordo & Katano, 2008). Considering how much plant phenology can vary between seasons, it seems that systematic sampling could be imperative for providing a reliable list of bee species for a specific area, and showing how much change can occur between seasons. Further work is necessary to evaluate if potential mismatches between food resources availability and adult native bee foraging periods can drive considerable losses of entomofauna for this high-altitude ecosystem. This is even more critical, considering that this work yielded a new species which is described in this paper, stressing the need for exploration and research on mountain ecosystems as the “final frontier” for many unknown species and their ecological interactions. Nonetheless, it is important to acknowledge that plant phenology still appears to be aligned to adult bee phenology, even though it has not been assessed whether changes have occurred over time (Arroyo, Armesto & Villagran, 1981; Henríquez-Piskulich et al., 2018).

## Conclusions

This work provides an updated list of native bee species and the phenology of each family of this high-altitude area for the flowering season, a list that was also compared with previously published articles regarding the ecosystems found at the High Andes of central Chile. Of the 58 native bee species reported for this area, this work was able to confirm the occurrence of 46 of these species. Halictids were the most abundant, and the only family differing in the peak of adult occurrence, which was during the month of December, while the remaining four families were mainly found in January.

The results of this study reinforce the importance of taxonomy and basic biology studies as major foundations for ecological research and conservation initiatives. To understand the role of each species in an ecosystem, the first step is to know what species are a part of it. These efforts are required to establish these foundations and are of utmost importance for the preservation of biodiversity, as well as, ultimately, our own survival (Thomson et al., 2018).

##  Supplemental Information

10.7717/peerj.8675/supp-1 Table S1 Full list of native bee species found in Farellones in previous surveys Each species identified in the three published works is indicated with an “X”.

10.7717/peerj.8675/supp-2 Table S2 Multiple comparisons for proportions of families This table shows post hoc comparison after Chi square on the proportion of specimens from different bee families found in our study, showing how different the proportions in the number of specimens collected were for each family during the 2017-2018 field survey, and if these differences were significant. First two columns correspond to the families being compared. This is followed by the difference, with values closer to zero representing less differences between the compared families, the q statistical value and significance at *p* < 0.05. Asterisks highlight if the difference in the proportion of specimens was significant or “NS” when not. ****

10.7717/peerj.8675/supp-3 Table S3 Multiple comparisons for proportions of genera This table shows post hoc comparison after Chi square on the proportion of specimens from different bee genera found in our study, showing how different the proportions in the number of specimens collected were for each genus during the 2017-2018 field survey, and if these differences were significant. First two columns correspond to the genera being compared. This is followed by the difference, with values closer to zero representing less differences between compared genera, the q statistical value and significance at *p* < 0.05. Asterisks highlight if the difference in the proportion of specimens was significant or “NS” when not.

10.7717/peerj.8675/supp-4 Table S4 Mardia-Watson-Wheeler uniform-scores test two-group hypothesis post hoc tests section This table shows if the families found in the study differed significantly in their temporal distribution, which is inferred from the date of collection of adult specimens belonging to each family during 2017-2018 field survey. First two columns show families that are being compared in each test. This is followed by statistical W value, where values closer to zero represent less differences in the distribution between compared groups. Significance at *p* < 0.05, is highlighted with an asterisk when significant or “NS” when not. ****

10.7717/peerj.8675/supp-5 Supplemental Information 1 Raw data used for the statistical analyses Every native bee specimen collected during the season 2016–2017 and 2017–2018 used for all statistical analyses in this work. Furthermore, relative abundance for both periods of collection have also been included.

## Additional Information and Declarations

### Data Availability

The following information was supplied regarding data availability: We deposited all type specimens at the entomological collection of the National Museum of Natural History, Santiago-Chile. Holotype: 8353⋅ Male: CHILE: REGIÓN METROPOLITANA: Santiago, Farellones, S33°22.084′, W070°16.264′, 2,465 m; pan traps, 2.xii.2017, P. Henríquez-Piskulich Paratypes: 8354⋅ Female: CHILE: REGIÓN METROPOLITANA: Santiago, Farellones, S33°22.084′, W070°16.264′, 2,465 m; pan traps, 2.xii.2017, P. Henríquez-Piskulich 8355⋅ Male: CHILE: REGIÓN METROPOLITANA: Santiago, Farellones, S33°22.084′, W070°16.264′, 2,465 m; pan traps, 2.ii.2018, P. Henríquez-Piskulich 8356⋅ Female: CHILE: REGIÓN METROPOLITANA: Santiago, Farellones, S33°22.084′, W070°16.264′, 2,465 m; pan traps, 2.ii.2018, P. Henríquez-Piskulich 8357⋅ Female: CHILE: REGIÓN METROPOLITANA: Santiago, Farellones, S33°22.084′, W070°16.264′, 2,465 m; pan traps, 2.ii.2018, P. Henríquez-Piskulich 8358⋅ Female: CHILE: REGIÓN METROPOLITANA: Santiago, Farellones, S33°22.084′, W070°16.264′, 2,465m; pan traps, 2.ii.2018, P. Henríquez-Piskulich

### New Species Registration

The following information was supplied regarding the registration of a newly described species: Publication LSID: urn:lsid:zoobank.org:pub:CAF131C8-3C76-49F0-87F9-76402590D913 Xeromelissa LSID: urn:lsid:zoobank.org:act:ED180AE3-648A-4F50-806A-CB9C6399F19E Xeromelissa sororitatis LSID: urn:lsid:zoobank.org:act:B0DC55A4-272F-4DB6-B311-D764B2448776

## References

[ref-1] Arroyo MTK, Armesto JJ, Villagran C (1981). Plant phenological patterns in the high Andean Cordillera of Central Chile. Journal of Ecology.

[ref-2] Arroyo MTK, Marquet PA, Simonetti JA, Cavieres LA, Mittermeier RA, Robles P, Hoffmann M, Pilgrim J, Brooks T, Mittermeier C, Lamoreux J, Da Fonseca GAB (2004). Chilean winter rainfall-Valdivian forests. Hotspots: earth’s biological richest and most endangered terrestrial ecoregions.

[ref-3] Arroyo MTK, Primack R, Armesto J (1982). Community studies in pollination ecology in the high temperate andes of Central Chile. I. Pollination mechanisms and altitudinal variation. American Journal of Botany.

[ref-4] Ascher JS, Pickering J (2019). Discover Life bee species guide and world checklist (Hymenoptera: Apoidea: Anthophila). http://www.discoverlife.org/mp/20q?guide=Apoidea_species.

[ref-5] Camousseight A, Barrera E (1998). Análisis Del Polen Transportado Por Insectos Estivales En El Sector De La Parva, Cordillera De Santiago. Revista Chilena de Entomología.

[ref-6] Chiappa E, Rojas M, Toro H (1990). Clave para los géneros de abejas de chile (Hymenoptera: Apoidea). Revista Chilena de Entomología.

[ref-7] Da Cruz DD, Mello MAR, Van Sluys M (2006). Phenology and floral visitors of two sympatric *Heliconia* species in the Brazilian Atlantic forest. Flora: Morphology, Distribution, Functional Ecology of Plants.

[ref-8] Doi H, Gordo O, Katano I (2008). Heterogeneous intra-annual climatic changes drive different phenological responses at two trophic levels. Climate Research.

[ref-9] Dos Santos SR, Specht A, Carneiro E, Vieira de Paula-Moraes SV, Martins M (2017). Interseasonal variation of *Chrysodeixis includens* (Walker, [1858]) (Lepidoptera: Noctuidae) populations in the Brazilian Savanna. Revista Brasileira de Entomologia.

[ref-10] Durante S, Abrahamovich A, Lucia M (2006). El Subgénero Megachile (Dasymegachile) Mitchell con Especial Referencia a las Especies Argentinas (Hymenoptera: Megachilidae). Neotropical Entomology.

[ref-11] Ferrari RR (2017). Taxonomic revision of the species of *Colletes* Latreille, 1802 (Hymenoptera: Colletidae: Colletinae) found in Chile. Zootaxa.

[ref-12] Fisher NI (1993). Statistical analysis of circular data.

[ref-13] Fortel L, Henry M, Guilbaud L, Guirao AL, Kuhlmann M, Mouret H, Rollin O, Vaissie BE (2014). Decreasing abundance, increasing diversity and changing structure of the wild bee community (Hymenoptera: Anthophila) along an urbanization gradient. PLOS ONE.

[ref-14] Freitas BM, Imperatriz-Fonseca VL, Medina LM, Peixoto Kleinert ADM, Galetto L, Nates-Parra G, Quezada-Euán JJG (2009). Diversity, threats and conservation of native bees in the Neotropics. Apidologie.

[ref-15] Frías-Lasserre D, Villagra CA (2019). Differences in larval emergence chronotypes for sympatric *Rhagoletis brncici* Frías and *Rhagoletis conversa* (Bréthes) (Diptera, Tephritidae). Revista Brasileira de Entomologia.

[ref-16] González E, Salvo A, Defagó MT, Valladares G (2016). A moveable feast: insects moving at the forest-crop interface are affected by crop phenology and the amount of forest in the landscape.

[ref-17] González VH, Engel MS (2004). The tropical andean Bee Fauna (Insecta: Hymenoptera: Apoidea), with examples from Colombia. Entomologische Abhandlungen.

[ref-18] González-Reyes AX, Corronca JA, Rodriguez-Artigas SM (2017). Changes of arthropod diversity across an altitudinal ecoregional zonation in Northwestern Argentina. PeerJ.

[ref-19] González-Vaquero RA, Galvani GL (2016). Antennal sensilla analyses as useful tools in the revision of the sweat-bee subgenus *Corynura* (Callistochlora) Michener (Hymenoptera: Halictidae). Zoologischer Anzeiger.

[ref-20] Gottfried M, Pauli H, Reiter K, Grabherr G (1999). A fine-scaled predictive model for changes in species distribution patterns of high mountain plants induced by climate warming. Diversity and Distributions.

[ref-21] Harris R (1979). A glossary of surface sculpturing. Ocassional papers in entomology 28.

[ref-22] Hegland SJ, Nielsen A, Lázaro A, Bjerknes AL, Totland Ø (2009). How does climate warming affect plant–pollinator interactions?. Ecology Letters.

[ref-23] Henríquez-Piskulich H, Vera A, Sandoval G, Villagra C (2018). Along urbanization sprawl, exotic plants distort native bee (Hymenoptera: Apoidea) assemblages in high elevation Andes ecosystem. PeerJ.

[ref-24] Hodges T (1990). Predicting crop phenology.

[ref-25] Hodkinson ID (2005). Terrestrial insects along elevation gradients: species and community responses to altitude. Biological Reviews.

[ref-26] Hoorn C, Mosbrugger V, Mulch A, Antonelli A (2013). Biodiversity from mountain building. Nature Publishing Group.

[ref-27] Jaccard P (1900). Etude de la distribution florale dans une portion des Alpes et du Jura. Bulletin de la Societe Vaudoise des Sciences Naturelles.

[ref-28] Kudo G, Ida TY (2013). Early onset of spring increases the phenological mismatch between plants and pollinators. Ecology.

[ref-29] Manuelli S, Hofer T, Springgay E (2019). FAO’s work in sustainable mountain development and watershed management—a 2017 update. Mountain Research and Development.

[ref-30] McCoy ED (1990). The distribution along elevational gradients of insects. Oikos.

[ref-31] McCravy KW, Ruholl JD (2017). Bee (Hymenoptera: Apoidea) diversity and sampling methodology in a midwestern USA deciduous forest. Insects.

[ref-32] Melo C, Scattolini C, Pocco ME, Montemayor SI, Scheibler EE, Roig SA (2017). The fate of endemic insects of the Andean region under the effect of global warming. PLOS ONE.

[ref-33] Memmott J, Craze PG, Waser NM, Price MV (2007). Global warming and the disruption of plant–pollinator interactions. Ecology Letters.

[ref-34] Miller DA, Nichols JD, MClintock BT, Grant EHC, Bailey LL, Weir LA (2013). Improving occupancy estimation when two types of observational error occur: non-detection and species misidentification. Ecology.

[ref-35] Monckton SK (2016). A revision of *Chilicola* (Heteroediscelis), a subgenus of xeromelissine bees (Hymenoptera, Colletidae) endemic to Chile: taxonomy, phylogeny, and biogeography, with descriptions of eight new species. ZooKeys.

[ref-36] Montalva J, Ruz L (2010). Actualización de la lista sistemática de las abejas chilenas (Hymenoptera: Apioidea). Revista Chilena de Entomología.

[ref-37] Morales C, Montalva J, Arbetman M, Aizen M, Smith-Ramírez C, Vieli L, Hatfield R (2016). Bombus dahlbomii.

[ref-38] Morellato LPC, Alberti LF, Hudson IL, Keatley ILH, MR (2010). Applications of circular statistics in plant phenology: a case studies approach. Phenological research: methods for environmental and climate change analysis.

[ref-39] Moure JS, Urban D (2012). Xeromelissini Cockerell, 1926. http://www.moure.cria.org.br/catalogue.

[ref-40] Murúa M, Cisterna J, Rosende B (2014). Pollination ecology and breeding system of two *Calceolaria* species in Chile. Revista Chilena De Historia Natural.

[ref-41] Myers N, Mittermeier RA, Mittermeier CG, Da Fonseca GAB, Kent J (2000). Biodiversity hotspots for conservation priorities. Nature.

[ref-42] Niemeyer H (2015). Hoyas Hidrográficas De Chile: Región Metropolitana.

[ref-43] Nürnberger F, Härtel S, Steffan-Dewenter I (2018). The influence of temperature and photoperiod on the timing of brood onset in hibernating honey bee colonies. PeerJ.

[ref-44] Ogilvie JE, Forrest JR (2017). Interactions between bee foraging and floral resource phenology shape bee populations and communities. Current Opinion in Insect Science.

[ref-45] Öztürk Ü, Hakeem KR, Faridah-Hanum I, Efe R (2015). Climate change impacts on high-altitude ecosystems.

[ref-46] Packer L (2008). Phylogeny and classification of the Xeromelissinae (Hymenoptera: Apoidea, Colletidae) with special emphasis on the genus *Chilicola*. Systematic Entomology.

[ref-47] Packer L (2019). Bee Genera of Chile. https://www.yorku.ca/bugsrus/resources/galleries/bgoc.

[ref-48] Packer L, Dumesh S (2014). Two new species of *Geodiscelis* Michener & Rozen (Hymenoptera: Apoidea: Colletidae) with a phylogenetic analysis and subgeneric classification of the genus. Zootaxa.

[ref-49] Packer L, Genaro JA (2007). Fifteen new species of *Chilicola* (Hymenoptera: Apoidea; Colletidae). Zootaxa.

[ref-50] Packer L, Monckton SK, Onuferko TM, Ferrari RR (2018). Validating taxonomic identifications in entomological research. Insect Conservation and Diversity.

[ref-51] Paknia O, Pfeiffer M (2011). Hierarchical partitioning of ant diversity: implications for conservation of biogeographical diversity in arid and semi-arid areas. Diversity and Distributions.

[ref-52] Palomo I (2017). Climate change impacts on ecosystem services in high mountain areas: a literature review. Mountain Research and Development.

[ref-53] Potts SG, Biesmeijer JC, Kremen C, Neumann P, Schweiger O, Kunin WE (2010). Global pollinator declines: trends, impacts and drivers. Trends in Ecology and Evolution.

[ref-54] Richardson DM, Whittaker RJ (2010). Conservation biogeography—foundations, concepts and challenges. Diversity and Distributions.

[ref-55] Robinet C, Roques A (2010). Direct impacts of recent climate warming on insect populations. Integrative Zoology.

[ref-56] Roig-Alsina A (1991). Revision of the Cleptoparasitic Bee Tribe Isepeolini (Hymenoptera: Anthophoridae). The University of Kansas Science Bulletin.

[ref-57] Roig-Alsina A (1999). Revision de las abejas colectoras de aeeites del género Chalepogenus Holmberg (Hymenoptera, Apiddae, Tapinotaspidini). Revista Museo Argentino Ciencias Naturales.

[ref-58] Rojas F (2001). Nueva Especie De *Ruizantheda* Moure (Apoidea: Halictidae: Halictini) De Chile. Revista Chilena de Entomología.

[ref-59] Rojas F, Toro H (2000). Revisión de las especies de *Caenohalictus* (Halictidae-Apoidea) Presentes en Chile. Boletín del Museo Nacional de Historia Natural, Chile.

[ref-60] Ruiz D, Moreno HA, Gutiérrez ME, Zapata PA (2008). Changing climate and endangered high mountain ecosystems in Colombia. Science of the Total Environment.

[ref-61] Ruz L, Chiappa E (2004). *Protandrena evansi*, a New Panurgine Bee from Chile (Hymenoptera: Andrenidae). Journal of the Kansas Entomological Society.

[ref-62] Ruz L, Toro H (1983). Revision of the Bee Genus *Liphanthus* (Hymenoptera: Andrenidae). The University of Kansas Science Bulletin.

[ref-63] Sánchez-Bayoa F, Wyckhuys KAG (2019). Worldwide decline of the entomofauna: a review of its drivers. Biological Conservation.

[ref-64] Sedano RE, Burns KJ (2010). Are the Northern Andes a species pump for Neotropical birds? Phylogenetics and biogeography of a clade of Neotropical tanagers (Aves: Thraupini). Journal of Biogeography.

[ref-65] Sielfeld W (1973). Contribución al conocimiento de las especies chilenas del género *Trichothurgus* Moure 1949 (Hymenoptera, Apoidea). Noticiario Mensual del Museo Nacional de Historia Natural.

[ref-66] Stiles FG (1975). Ecology, Flowering phenology, and hummingbird pollination of some Costa Rican *Heliconia* species. Ecology.

[ref-67] Tang J, Körner C, Muraoka H, Piao S, Shen M, Thackeray SJ, Yang X (2016). Emerging opportunities and challenges in phenology: a review. Ecosphere.

[ref-68] Taylor D, Kent M, Coker P (1993). Vegetation description and analysis: a practical approach. The Geographical Journal.

[ref-69] Theobald EJ, Breckheimer I, HilleRisLambers J (2017). Climate drives phenological reassembly of a mountain wildflower meadow community. Ecology.

[ref-70] Thomson SA, Ahyong ST, Alonso-Zarazaga M, Ammirati J, Araya JF, Pyle RL, Bailly N, Baker WJ, Balke M, Maxwell V, Elı GD, Dickinson C, Dickinson TA, Van Dijk PP, Dijkstra KB, Grazziotin G, Greenslade P, Hazevoet CJ, He K, He X, Helfer S, Helgen KM, Papp V, Parenti LR, Patterson D, Pavlinov IY, Todd JA, Triebel D, Winston JE, Wu W (2018). Taxonomy based on science is necessary for global conservation. PLOS Biology.

[ref-71] Toro H, Moldenke A (1979). Revisión De Los Xeromelissinae Chilenos (Hymenoptera—Colletidae). Anales Del Museo De Historia Natural.

[ref-72] Toro H, Rodríguez S (1998). Los Anthidiini De Chile: Clave Para Especies (Himenoptera: Megachilidae). Acta Entomológica Chilena.

[ref-73] Toro H, Ruz L (1969). Contribución Al Conocimiento Del Genero *Diadasia* (Hymenoptera-Anthophoridae) En Chile. Anales Del Museo De Historia Natural.

[ref-74] Tucker EM, Rehan SM (2017). High elevation refugia for *Bombus terricola* (Hymenoptera: Apidae) conservation and wild bees of the White Mountain national forest. Journal of Insect Science.

[ref-75] Vivallo F (2009). Notes on the bee genus *Alloscirtetica* Holmberg, 1909 in northern Chile with the description of two new altiplanic species and a key for the Chilean species of Eucerini (Hymenoptera: Apidae). Zootaxa.

[ref-76] Vivallo F (2013). Revision of the bee subgenus *Centris* (Wagenknechtia) Moure, 1950 (Hymenoptera: Apidae: Centridini). Zootaxa.

[ref-77] Vivallo F (2014). Taxonomic revision of the cleptoparasitic bee genus *Epiclopus* Spinola, 1851 (Hymenoptera: Apidae: Ericrocidini). Zootaxa.

[ref-78] Vivallo F, Zanella FCV, Toro H, Melo GAR, Alves-dos-Santos I (2003). Las especies chilenas de *Centris* (Paracentris) Cameron y *Centris* (Penthemisia) Moure (Hymenoptera, Apidae). Apoidea Neotropica: Homenagem aos 90 Anos de Jesus Santiago Moure.

[ref-79] Wadgymar SM, Ogilvie JE, Inouye DW, Weis AE, Anderson JT (2018). Phenological responses to multiple environmental drivers under climate change: insights from a long-term observational study and a manipulative field experiment. New Phytologist.

[ref-80] Westphal C, Bommarco R, Carré G, Lamborn E, Morison N, Petanidou T, Potts SG, Roberts SPM, Szentgyörgyi H, Tscheulin T, Vaissière BE, Woyciechowski M, Biesmeuer JC, Kunin WE, Settele J, Steffan-Dewenter I (2008). Measuring bee diversity in different European habitats and biogeographical regions. Ecological Monographs.

[ref-81] Wilson JS, Forister ML, Carril OM (2017). Interest exceeds understanding in public support of bee conservation. Frontiers in Ecology and the Environment.

[ref-82] Wilson JS, Wilson LE, Loftis LD, Griswold T (2010). The Montane Bee Fauna of North Central Washington, USA, with Floral Associations. Western North American Naturalist.

[ref-83] WWF (2019). Ecoregions. https://www.worldwildlife.org/biomes.

[ref-84] Zar J (1999). Biostatistical analysis.

