# Supplementary material for: Native bees of high Andes of Central Chile (Hymenoptera: Apoidea): biodiversity, phenology and the description of a new species of Xeromelissa Cockerell (Hymenoptera: Colletidae: Xeromelissinae)
Source: PeerJ. 2020 Feb 28;8:e8675. doi: 10.7717/peerj.8675 (PMC7050550; doi:10.7717/peerj.8675)
Supplement: Table S1 — Each species identified in the three published works is indicated with an “X”. [file peerj-08-8675-s001.docx]

| **Family**  Species | Survey 2016-2018* | Camousseight & Barrera 1998 | Arroyo *et al.* 1982 |
| --- | --- | --- | --- |
| **Andrenidae** | | | |
| *Acamptopoeum submetallicum* | X |  |  |
| *Euherbstia excellens* | X |  |  |
| *Liphanthus andinus* | X |  |  |
| *Liphanthus coquimbensis* | X |  |  |
| *Liphanthus sabulosus* | X |  | X |
| *Liphanthus* spp. |  |  | X |
| *Rhophitulus evansi* | X |  |  |
| **Colletidae** | | | |
| *Cadeguala occidentalis* | X |  | X |
| *Caupolicana bicolor* | X |  |  |
| *Caupolicana dimidiata* |  |  | X |
| *Chilicola curvapeligrosa* | X |  |  |
| *Chilicola* spp. |  |  | X |
| *Colletes araucariae* |  | X | X |
| *Colletes kuhlmanni* | X |  |  |
| *Colletes fulvipes* | X | X | X |
| *Colletes musculus* | X |  |  |
| *Colletes* sp. |  |  | X |
| *Mourecotelles ruizii* |  |  | X |
| *Xanthocotelles incahuasi* |  |  | X |
| *Xanthocotelles sicheli* |  |  | X |
| *Xeromelissa farellones* |  |  |  |
| *Xeromelissa sororitatis*, **n. sp.**  **++** | X |  | X |
| **Halictidae** | | | |
| *Caenohalictus aplacodes* | X |  |  |
| *Caenohalictus iodurus* | X | X |  |
| *Caenohalictus rostraticeps* | X |  |  |
| *Caenohalictus* spp. |  |  | X |
| *Callistochlora chloris* | X |  |  |
| *Callistochlora prothysteres* | X |  |  |
| *Corynura* sp. |  |  | X |
| *Lasioglossum* (*Dialictus*) spp. | X |  | X |
| *Ruizantheda cerdai* | X |  |  |
| *Ruizantheda mutabilis* | X |  | X |
| *Ruizantheda nigrocaerulea* | X |  |  |
| *Ruizantheda proxima* | X |  |  |
| *Sphecodes granulosus* | X |  |  |
| **Megachilidae** | | | |
| *Anthidium chilense* | X |  | X |
| *Anthidium chubuti* | X |  |  |
| *Anthidium decaspilum* |  | X | X |
| *Anthidium espinosai* | X |  | X |
| *Anthidium funereum* |  |  | X |
| *Anthidium gayi* |  |  | X |
| *Anthidium rubripes* |  |  | X |
| *Anthidium* sp*.* |  |  | X |
| *Coelioxys* sp. |  |  | X |
| *Megachile distinguenda* | X |  |  |
| *Megachile pollinosa* | X | X |  |
| *Megachile saulcyi* | X |  | X |
| *Megachile semirufa* | X | X | X |
| *Megachile* spp*.* |  |  | X |
| *Trichothurgus herbsti* | X |  |  |
| *Trichothurgus wagenknechti* |  |  | X |
| **Apidae** | | | |
| *Alloscirtetica gayi* | X |  | X |
| *Alloscirtetica rufitarsis* | X | X | X |
| *Alloscirtetica* sp. |  |  | X |
| *Anthophora incerta* | X |  | X |
| *Anthophora* sp. |  |  | X |
| *Apis mellifera* | X |  |  |
| *Bombus dahlbomii* | X |  | X |
| *Bombus terrestris* | X |  |  |
| *Centris cineraria* | X | X | X |
| *Centris nigerrima* | X | X | X |
| *Chalepogenus caeruleus* | X |  | X |
| *Chalepogenus herbsti* |  | X | X |
| *Chalepogenus* sp. |  | X |  |
| *Diadasia chilensis* | X |  |  |
| *Epiclopus gayi* | X |  | X |
| *Epiclopus lendlianus* | X | X |  |
| *Isepeolus luctuosus* | X |  |  |
| *Melectoides niveiventris* |  |  | X |
| *Svastrides melanura* | X |  | X |

*January 2019 was also sampled for this survey.

++Described as *Xeromelissa* sp. in Henríquez-Piskulich et al. 2018.
