# Supplementary material for: Native bees of high Andes of Central Chile (Hymenoptera: Apoidea): biodiversity, phenology and the description of a new species of Xeromelissa Cockerell (Hymenoptera: Colletidae: Xeromelissinae)
Source: PeerJ. 2020 Feb 28;8:e8675. doi: 10.7717/peerj.8675 (PMC7050550; doi:10.7717/peerj.8675)
Supplement: Table S2 — This table shows post hoc comparison after Chi square on the proportion of specimens from different bee families found in our study, showing how different the proportions in the number of specimens collected were for each family during the 2017-2018 field survey, and if these differences were significant. First two columns correspond to the families being compared. This is followed by the difference, with values closer to zero representing less differences between the compared families, the q statistical value and significance at p < 0.05. Asterisks highlight if the difference in the proportion of specimens was significant or “NS” when not. [file peerj-08-8675-s002.docx]

|  |  |  |  |  |  |  |  |
| --- | --- | --- | --- | --- | --- | --- | --- |
|  |  |  |  |  |  |  |  |
|  | **Multiple Comparisons for Proportions of Families** | | |  |  |  |  |
|  |  |  |  |  |  |  |  |
|  |  | **vs.** | **Diff** | **q** | ***p*** |  |  |
|  |  |  |  | q(0.05)=3.86 |  |  |  |
|  |  |  |  |  |  |  |  |
|  |  |  |  |  |  |  |  |
|  | **Halictidae** | Megachilidae | 30.87 | 31.24 | <0.001 | ** |  |
|  |  | Colletidae | 25.84 | 26.15 | <0.001 | ** |  |
|  |  | Andrenidae | 21.03 | 21.28 | <0.001 | ** |  |
|  |  | Apidae | 17.56 | 17.77 | <0.001 | ** |  |
|  | **Apidae** | Megachilidae | 13.31 | 13.47 | <0.001 | ** |  |
|  |  | Colletidae | 8.28 | 8.38 | <0.01 | * |  |
|  |  | Andrenidae | 3.47 | 3.51 | >0.05 | NS |  |
|  | **Andrenidae** | Megachilidae | 9.84 | 9.96 | <0.01 | * |  |
|  |  | Colletidae | 4.81 | 4.87 | <0.05 | * |  |
|  | **Colletidae** | Megachilidae | 5.03 | 5.09 | <0.05 | * |  |
|  |  |  |  |  |  |  |  |
|  |  |  |  |  |  |  |  |
