# Supplementary material for: Native bees of high Andes of Central Chile (Hymenoptera: Apoidea): biodiversity, phenology and the description of a new species of Xeromelissa Cockerell (Hymenoptera: Colletidae: Xeromelissinae)
Source: PeerJ. 2020 Feb 28;8:e8675. doi: 10.7717/peerj.8675 (PMC7050550; doi:10.7717/peerj.8675)
Supplement: Table S3 — This table shows post hoc comparison after Chi square on the proportion of specimens from different bee genera found in our study, showing how different the proportions in the number of specimens collected were for each genus during the 2017-2018 field survey, and if these differences were significant. First two columns correspond to the genera being compared. This is followed by the difference, with values closer to zero representing less differences between compared genera, the q statistical value and significance at p < 0.05. Asterisks highlight if the difference in the proportion of specimens was significant or “NS” when not. [file peerj-08-8675-s003.docx]

|  |  |  |  |  |  |  |  |  |
| --- | --- | --- | --- | --- | --- | --- | --- | --- |
|  |  |  |  |  |  |  |  |  |
|  |  | **Multiple Comparisons for Proportions of Genera** | | |  |  |  |  |
|  |  |  |  |  |  |  |  |  |
|  |  |  | **vs.** | **Diff** | **q** | ***p*** |  |  |
|  |  |  |  |  | q(0.05)=4.89 |  |  |  |
|  |  |  |  |  |  |  |  |  |
|  |  |  |  |  |  |  |  |  |
|  | 1 | *Lasioglossum* | *Protandrena* | 28.10 | 28.18 | <0.001 | ** |  |
|  |  |  | *Ruizantheda* | 28.10 | 28.18 | <0.001 | ** |  |
|  |  |  | *Alloscirtetica* | 27.75 | 27.83 | <0.001 | ** |  |
|  |  |  | *Xeromelissa* | 27.41 | 27.49 | <0.001 | ** |  |
|  |  |  | *Anthidium* | 26.50 | 26.58 | <0.001 | ** |  |
|  |  |  | *Rhophitulus* | 25.69 | 25.77 | <0.001 | ** |  |
|  |  |  | *Diadasia* | 25.69 | 25.77 | <0.001 | ** |  |
|  |  |  | *Acamptopoeum* | 24.50 | 24.57 | <0.001 | ** |  |
|  |  |  | *Centris* | 23.86 | 23.93 | <0.001 | ** |  |
|  |  |  | *Chilicola* | 22.67 | 22.74 | <0.001 | ** |  |
|  |  |  | *Megachile* | 21.95 | 22.01 | <0.001 | ** |  |
|  |  |  | *Colletes* | 20.14 | 20.20 | <0.001 | ** |  |
|  |  |  | *Callistochlora* | 17.60 | 17.65 | <0.001 | ** |  |
|  |  |  | *Liphanthus* | 14.46 | 14.50 | <0.001 | ** |  |
|  |  |  | *Caenohalictus* | 14.46 | 14.50 | <0.001 | ** |  |
|  |  |  | *Bombus* | 11.81 | 11.84 | <0.001 | ** |  |
|  | 2 | *Bombus* | *Protandrena* | 16.29 | 16.34 | <0.001 | ** |  |
|  |  |  | *Ruizantheda* | 16.29 | 16.34 | <0.001 | ** |  |
|  |  |  | *Alloscirtetica* | 15.94 | 15.98 | <0.001 | ** |  |
|  |  |  | *Xeromelissa* | 15.60 | 15.65 | <0.001 | ** |  |
|  |  |  | *Anthidium* | 14.69 | 14.74 | <0.001 | ** |  |
|  |  |  | *Rhophitulus* | 13.89 | 13.93 | <0.001 | ** |  |
|  |  |  | *Diadasia* | 13.89 | 13.93 | <0.001 | ** |  |
|  |  |  | *Acamptopoeum* | 12.69 | 12.73 | <0.001 | ** |  |
|  |  |  | *Centris* | 12.05 | 12.08 | <0.001 | ** |  |
|  |  |  | *Chilicola* | 10.87 | 10.90 | <0.001 | ** |  |
|  |  |  | *Megachile* | 10.14 | 10.17 | <0.001 | ** |  |
|  |  |  | *Colletes* | 8.34 | 8.36 | <0.01 | * |  |
|  |  |  | *Callistochlora* | 5.79 | 5.81 | <0.01 | * |  |
|  |  |  | *Liphanthus* | 2.65 | 2.66 | ≥0.05 | NS |  |
|  |  |  | *Caenohalictus* | 2.65 | 2.66 | ≥0.05 | NS |  |
|  | 3 | *Caenohalictus* | *Protandrena* | 13.64 | 13.68 | <0.01 | * |  |
|  |  |  | *Ruizantheda* | 13.64 | 13.68 | <0.001 | ** |  |
|  |  |  | *Alloscirtetica* | 13.29 | 13.33 | <0.001 | ** |  |
|  |  |  | *Xeromelissa* | 12.95 | 12.99 | <0.001 | ** |  |
|  |  |  | *Anthidium* | 12.04 | 12.08 | <0.001 | ** |  |
|  |  |  | *Rhophitulus* | 11.23 | 11.27 | <0.001 | ** |  |
|  |  |  | *Diadasia* | 11.23 | 11.27 | <0.001 | ** |  |
|  |  |  | *Acamptopoeum* | 10.04 | 10.07 | <0.001 | ** |  |
|  |  |  | *Centris* | 9.40 | 9.42 | <0.001 | ** |  |
|  |  |  | *Chilicola* | 8.21 | 8.24 | <0.001 | ** |  |
|  |  |  | *Megachile* | 7.49 | 7.51 | <0.01 | * |  |
|  |  |  | *Colletes* | 5.69 | 5.70 | <0.01 | * |  |
|  |  |  | *Callistochlora* | 3.14 | 3.15 | ≥0.05 | NS |  |
|  |  |  | *Liphanthus* | 0 | 0 | ≥0.05 | NS |  |
|  | 4 | *Liphanthus* | *Protandrena* | 13.64 | 13.68 | <0.001 | ** |  |
|  |  |  | *Ruizantheda* | 13.64 | 13.68 | <0.001 | ** |  |
|  |  |  | *Alloscirtetica* | 13.29 | 13.33 | <0.001 | ** |  |
|  |  |  | *Xeromelissa* | 12.95 | 12.99 | <0.001 | ** |  |
|  |  |  | *Anthidium* | 12.04 | 12.08 | <0.001 | ** |  |
|  |  |  | *Rhophitulus* | 11.23 | 11.27 | <0.001 | ** |  |
|  |  |  | *Diadasia* | 11.23 | 11.27 | <0.001 | ** |  |
|  |  |  | *Acamptopoeum* | 10.04 | 10.07 | <0.001 | ** |  |
|  |  |  | *Centris* | 9.40 | 9.42 | <0.01 | * |  |
|  |  |  | *Chilicola* | 8.21 | 8.24 | <0.01 | * |  |
|  |  |  | *Megachile* | 7.49 | 7.51 | <0.01 | * |  |
|  |  |  | *Colletes* | 5.69 | 5.70 | <0.01 | * |  |
|  |  |  | *Callistochlora* | 3.14 | 3.15 | ≥0.05 | NS |  |
|  | 5 | *Callistochlora* | *Protandrena* | 10.50 | 10.53 | <0.001 | ** |  |
|  |  |  | *Ruizantheda* | 10.50 | 10.53 | <0.001 | ** |  |
|  |  |  | *Alloscirtetica* | 10.15 | 10.18 | <0.001 | ** |  |
|  |  |  | *Xeromelissa* | 9.81 | 9.84 | <0.001 | ** |  |
|  |  |  | *Anthidium* | 8.90 | 8.93 | <0.001 | ** |  |
|  |  |  | *Rhophitulus* | 8.09 | 8.12 | <0.001 | ** |  |
|  |  |  | *Diadasia* | 8.09 | 8.12 | <0.001 | ** |  |
|  |  |  | *Acamptopoeum* | 6.90 | 6.92 | <0.01 | * |  |
|  |  |  | *Centris* | 6.26 | 6.27 | <0.01 | * |  |
|  |  |  | *Chilicola* | 5.07 | 5.09 | <0.05 | * |  |
|  |  |  | *Megachile* | 4.35 | 4.36 | ≥0.05 | NS |  |
|  |  |  | *Colletes* | 2.55 | 2.55 | ≥0.05 | NS |  |
|  | 6 | *Colletes* | *Protandrena* | 7.96 | 7.98 | <0.001 | ** |  |
|  |  |  | *Ruizantheda* | 7.96 | 7.98 | <0.001 | ** |  |
|  |  |  | *Alloscirtetica* | 7.60 | 7.62 | <0.001 | ** |  |
|  |  |  | *Xeromelissa* | 7.27 | 7.29 | <0.001 | ** |  |
|  |  |  | *Anthidium* | 6.36 | 6.37 | <0.01 | * |  |
|  |  |  | *Rhophitulus* | 5.55 | 5.56 | <0.05 | * |  |
|  |  |  | *Diadasia* | 5.55 | 5.56 | <0.05 | * |  |
|  |  |  | *Acamptopoeum* | 4.36 | 4.37 | ≥0.05 | NS |  |
|  |  |  | *Centris* | 3.71 | 3.72 | ≥0.05 | NS |  |
|  |  |  | *Chilicola* | 2.53 | 2.54 | ≥0.05 | NS |  |
|  |  |  | *Megachile* | 1.80 | 1.81 | ≥0.05 | NS |  |
|  | 7 | *Megachile* | *Protandrena* | 6.15 | 6.17 | <0.05 | * |  |
|  |  |  | *Ruizantheda* | 6.15 | 6.17 | <0.05 | * |  |
|  |  |  | *Alloscirtetica* | 5.80 | 5.82 | <0.05 | * |  |
|  |  |  | *Xeromelissa* | 5.46 | 5.48 | <0.01 | * |  |
|  |  |  | *Anthidium* | 4.55 | 4.57 | ≥0.05 | NS |  |
|  |  |  | *Rhophitulus* | 3.75 | 3.76 | ≥0.05 | NS |  |
|  |  |  | *Diadasia* | 3.75 | 3.76 | ≥0.05 | NS |  |
|  |  |  | *Acamptopoeum* | 2.55 | 2.56 | ≥0.05 | NS |  |
|  |  |  | *Centris* | 1.91 | 1.91 | ≥0.05 | NS |  |
|  |  |  | *Chilicola* | 0.72 | 0.73 | ≥0.05 | NS |  |
|  | 8 | *Chilicola* | *Protandrena* | 5.43 | 5.44 | <0.05 | * |  |
|  |  |  | *Ruizantheda* | 5.43 | 5.44 | <0.05 | * |  |
|  |  |  | *Alloscirtetica* | 5.07 | 5.09 | <0.01 | * |  |
|  |  |  | *Xeromelissa* | 4.74 | 4.75 | ≥0.05 | NS |  |
|  |  |  | *Anthidium* | 3.83 | 3.84 | ≥0.05 | NS |  |
|  |  |  | *Rhophitulus* | 3.02 | 3.03 | ≥0.05 | NS |  |
|  |  |  | *Diadasia* | 3.02 | 3.03 | ≥0.05 | NS |  |
|  |  |  | *Acamptopoeum* | 1.83 | 1.84 | ≥0.05 | NS |  |
|  |  |  | *Centris* | 1.18 | 1.19 | ≥0.05 | NS |  |
|  | 9 | *Centris* | *Protandrena* | 4.25 | 4.26 | ≥0.05 | NS |  |
|  |  |  | *Ruizantheda* | 4.25 | 4.26 | ≥0.05 | NS |  |
|  |  |  | *Alloscirtetica* | 3.89 | 3.90 | ≥0.05 | NS |  |
|  |  |  | *Xeromelissa* | 3.56 | 3.57 | ≥0.05 | NS |  |
|  |  |  | *Anthidium* | 2.65 | 2.65 | ≥0.05 | NS |  |
|  |  |  | *Rhophitulus* | 1.84 | 1.84 | ≥0.05 | NS |  |
|  |  |  | *Diadasia* | 1.84 | 1.84 | ≥0.05 | NS |  |
|  |  |  | *Acamptopoeum* | 0.65 | 0.65 | ≥0.05 | NS |  |
|  | 10 | *Acamptopoeum* | *Protandrena* | 3.60 | 3.61 | ≥0.05 | NS |  |
|  |  |  | *Ruizantheda* | 3.60 | 3.61 | ≥0.05 | NS |  |
|  |  |  | *Alloscirtetica* | 3.24 | 3.25 | ≥0.05 | NS |  |
|  |  |  | *Xeromelissa* | 2.91 | 2.92 | ≥0.05 | NS |  |
|  |  |  | *Anthidium* | 2.00 | 2.00 | ≥0.05 | NS |  |
|  |  |  | *Rhophitulus* | 1.19 | 1.19 | ≥0.05 | NS |  |
|  |  |  | *Diadasia* | 1.19 | 1.19 | ≥0.05 | NS |  |
|  | 11 | *Diadasia* | *Protandrena* | 2.41 | 2.41 | ≥0.05 | NS |  |
|  |  |  | *Ruizantheda* | 2.41 | 2.41 | ≥0.05 | NS |  |
|  |  |  | *Alloscirtetica* | 2.05 | 2.06 | ≥0.05 | NS |  |
|  |  |  | *Xeromelissa* | 1.72 | 1.72 | ≥0.05 | NS |  |
|  |  |  | *Anthidium* | 0.81 | 0.81 | ≥0.05 | NS |  |
|  |  |  | *Rhophitulus* | 0 | 0 | ≥0.05 | NS |  |
|  | 12 | *Rhophitulus* | *Protandrena* | 2.41 | 2.41 | ≥0.05 | NS |  |
|  |  |  | *Ruizantheda* | 2.41 | 2.41 | ≥0.05 | NS |  |
|  |  |  | *Alloscirtetica* | 2.05 | 2.06 | ≥0.05 | NS |  |
|  |  |  | *Xeromelissa* | 1.72 | 1.72 | ≥0.05 | NS |  |
|  |  |  | *Anthidium* | 0.81 | 0.81 | ≥0.05 | NS |  |
|  | 13 | *Anthidium* | *Protandrena* | 1.60 | 1.60 | ≥0.05 | NS |  |
|  |  |  | *Ruizantheda* | 1.60 | 1.60 | ≥0.05 | NS |  |
|  |  |  | *Alloscirtetica* | 1.25 | 1.25 | ≥0.05 | NS |  |
|  |  |  | *Xeromelissa* | 0.91 | 0.91 | ≥0.05 | NS |  |
|  | 14 | *Xeromelissa* | *Protandrena* | 0.69 | 0.69 | ≥0.05 | NS |  |
|  |  |  | *Ruizantheda* | 0.69 | 0.69 | ≥0.05 | NS |  |
|  |  |  | *Alloscirtetica* | 0.33 | 0.34 | ≥0.05 | NS |  |
|  | 15 | *Alloscirtetica* | *Protandrena* | 0.35 | 0.36 | ≥0.05 | NS |  |
|  |  |  | *Ruizantheda* | 0.35 | 0.36 | ≥0.05 | NS |  |
|  |  |  | *Alloscirtetica* | 0 | 0 | ≥0.05 | NS |  |
|  |  |  |  |  |  |  |  |  |
|  |  |  |  |  |  |  |  |  |
|  |  |  |  |  |  |  |  |  |
