# Supplementary material for: Native bees of high Andes of Central Chile (Hymenoptera: Apoidea): biodiversity, phenology and the description of a new species of Xeromelissa Cockerell (Hymenoptera: Colletidae: Xeromelissinae)
Source: PeerJ. 2020 Feb 28;8:e8675. doi: 10.7717/peerj.8675 (PMC7050550; doi:10.7717/peerj.8675)
Supplement: Table S4 — This table shows if the families found in the study differed significantly in their temporal distribution, which is inferred from the date of collection of adult specimens belonging to each family during 2017-2018 field survey. First two columns show families that are being compared in each test. This is followed by statistical W value, where values closer to zero represent less differences in the distribution between compared groups. Significance at p < 0.05, is highlighted with an asterisk when significant or “NS” when not. [file peerj-08-8675-s004.docx]

|  |  |  |  |  |  |  |
| --- | --- | --- | --- | --- | --- | --- |
|  |  |  |  |  |  |  |
|  | **First** | **Second** | **Equal Distributions** | ***p*** |  |  |
|  | **Family** | **Family** | **Test *W*** |  |  |  |
|  |  |  |  |  |  |  |
|  | Andrenidae | Colletidae | 8.84 | 0.01 | * |  |
|  | Andrenidae | Halictidae | 142.55 | 0 | * |  |
|  | Andrenidae | Megachilidae | 6.66 | 0.04 | * |  |
|  | Andrenidae | Apidae | 7.22 | 0.03 | * |  |
|  | Colletidae | Halictidae | 86.27 | 0 | * |  |
|  | Colletidae | Megachilidae | 5.84 | 0.05 | NS |  |
|  | Colletidae | Apidae | 4.12 | 0.13 | NS |  |
|  | Halictidae | Megachilidae | 108.29 | 0 | * |  |
|  | Halictidae | Apidae | 166.99 | 0 | * |  |
|  | Megachilidae | Apidae | 5.46 | 0.07 | NS |  |
|  |  |  |  |  |  |  |
|  |  |  |  |  |  |  |
